# Lupane Triterpenes with Antileukemia Activity from *Maytenus quadrangulata*

**DOI:** 10.1021/acsomega.5c13073

**Published:** 2026-03-27

**Authors:** Sandy V. M. Quintão, Mariana G. de Aguilar, Lucas C. Souza, Túlio R. Freitas, Maria E. C. dos S. Jardim, Lohanne B. E. de Souza, Adriano de P. Sabino, Lucienir P. Duarte, Grasiely F. de Sousa

**Affiliations:** † Departamento de Química, ICEx, Universidade Federal de Minas Gerais, Belo Horizonte 31270-901, MG, Brazil; ‡ Departamento de Análises Clínicas e Toxicológicas, Faculdade de Farmácia, Universidade Federal de Minas Gerais, Belo Horizonte 31270-901, MG, Brazil

## Abstract

*Maytenus quadrangulata* occurs naturally in the Brazilian biomes Caatinga and Atlantic Forest, and previous studies have demonstrated its potential as a source of bioactive molecules. In light of this, a phytochemical study of the chloroform extract of *Maytenus quadrangulata* branches was carried out, yielding the new triterpene 30-oxo-2,3-*seco*-lup-20­(29)-ene-2,3-dioic acid (**1**) and a mixture containing the new semisynthetic triterpene 25-hydroxy-7-oxofriedelan-3α-yl acetate (**2**). In addition, seven known compounds were also obtained: friedelan-3-one (**3**), friedelan-3β-ol (**4**), friedelan-3α-ol (**5**), a mixture of long-chain fatty acids of β-sitosterol and lupeol-β-sitosterol alkanoate (**6**) and lupeol alkanoate (**7**), friedelane-3,7-dione (**8**) and 2,3-*seco*-lup-20­(29)-ene-2,3-dioic acid (**9**). Structural elucidation of the isolated metabolites was performed using ^1^H and ^13^C nuclear magnetic resonance (NMR). The chemical structures of the new compounds, **1** and **2**, were confirmed by the analysis of two-dimensional NMR spectra (HSQC, HMBC, COSY, and NOESY). An *in vitro* cytotoxicity assay against the THP-1 and K-562 leukemia cell lines and an *in silico* analysis using ADMETLab 3.0 were performed for compounds **1** and **9**. The novel triterpene (**1**) exhibited the highest cytotoxicity and the most favorable drug-like profile, underscoring its potential as an anticancer therapeutic.

## Graphical abstract

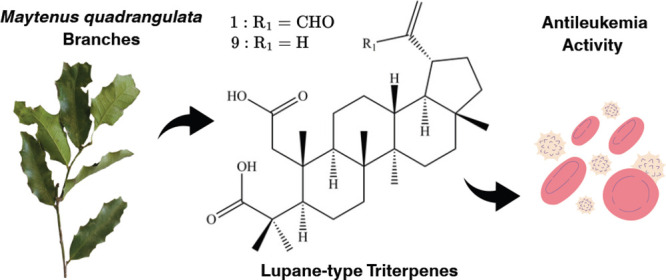

## Introduction

The Celastraceae family comprises 94 genera and approximately 1410 species, distributed throughout the subtropical and tropical regions of the planet.^1^ The ethnopharmacological use of these plants in traditional medicine underscores their relevance as targets for phytochemical studies. *Maytenus*, one of the largest genera in this family, is composed of approximately 300 species, including *M. elaeodendroides*, traditionally used in Cuba to treat inflammatory processes, and *M. senegalensis*, popularly employed for the treatment of malaria in Tanzania.^2,3^ In addition, *M. emarginata* has been utilized in India to treat a range of conditions, including fever, nervous disorders, allergy, microbial infection, mouth ulcers, and rheumatoid arthritis.^4^ Moreover, the ethanolic extract of the roots of *M. putterlickoides* displays antileukemic activity; the methanolic and ethyl acetate extracts of *M. truncata* leaves exhibit analgesic and antiulcer effects; and the ethanolic extract of *M. rigida* leaves possess antidiarrheal, antiulcer, and anti-inflammatory properties.^5^ The dry extract of *M. ilicifolia* leaves constitutes the active ingredient of the herbal medicine “Espinheira Santa,” used as a gastric protective agent in Brazil.^6^ Additionally, a recent study demonstrated that the ethanolic extract of this species and its organic fractions exhibit inhibitory effects on glycation and lipid peroxidation, present antioxidant and antimicrobial properties, and inhibit enzymes associated with diabetes and Alzheimer’s disease.^7^

*Maytenus quadrangulata* (Schrad.) Loes (synonym *Monteverdia quadrangulata*) is found in the Brazilian biomes Caatinga and Atlantic Forest, and is popularly known as “espinho-de-deus.”^8^ The first study of this species was performed on the leaves by Aguilar et al., which revealed its cytotoxic potential. The hexane extract, along with triterpenes isolated from it, was tested against leukemia, breast, and ovarian cancer cell lines.^9^ Furthermore, the antiviral activity of the ethyl acetate extracts from the leaves and branches was evaluated against Mayaro virus, and compounds isolated from the ethyl acetate extract from its leaves were tested against *Staphylococcus aureus* and *Klebsiella pneumoniae*.^10,11^ These results encourage further research on this species to uncover novel bioactive molecules. Therefore, in this work, the phytochemical study of the chloroform extract of *M. quadrangulata* branches was carried out, leading to the isolation of the new triterpene 30-oxo-2,3-*seco*-lup-20­(29)-ene-2,3-dioic acid (**1**) and a mixture containing the new semisynthetic triterpene 25-hydroxy-7-oxofriedelan-3α-yl acetate (**2**). In addition, seven other compounds previously reported in the literature were obtained: friedelan-3-one (**3**), friedelan-3β-ol (**4**), friedelan-3α-ol (**5**), a mixture of β-sitosterol alkanoate (**6**) and lupeol alkanoate (**7**), friedelane-3,7-dione (**8**) and 2,3-*seco*-lup-20­(29)-ene-2,3-dioic acid (**9**) (Figure 1). The structural elucidation was performed using infrared (IR) spectroscopy, ^1^H and ^13^C nuclear magnetic resonance (NMR), including two-dimensional NMR (HSQC, HMBC, COSY, and NOESY), and high-resolution electrospray ionization mass spectrometry (HR-ESI-MS). The cytotoxicity of compounds **1** and **9** was evaluated against the leukemia cell lines, THP-1 and K-562. Furthermore, an *in silico* ADMET (Absorption, Distribution, Metabolism, Excretion, and Toxicity) analysis was conducted for compounds **1** and **9** using the online platform ADMETLab 3.0 to assess their physicochemical and pharmacokinetic properties.

**Figure 1:**
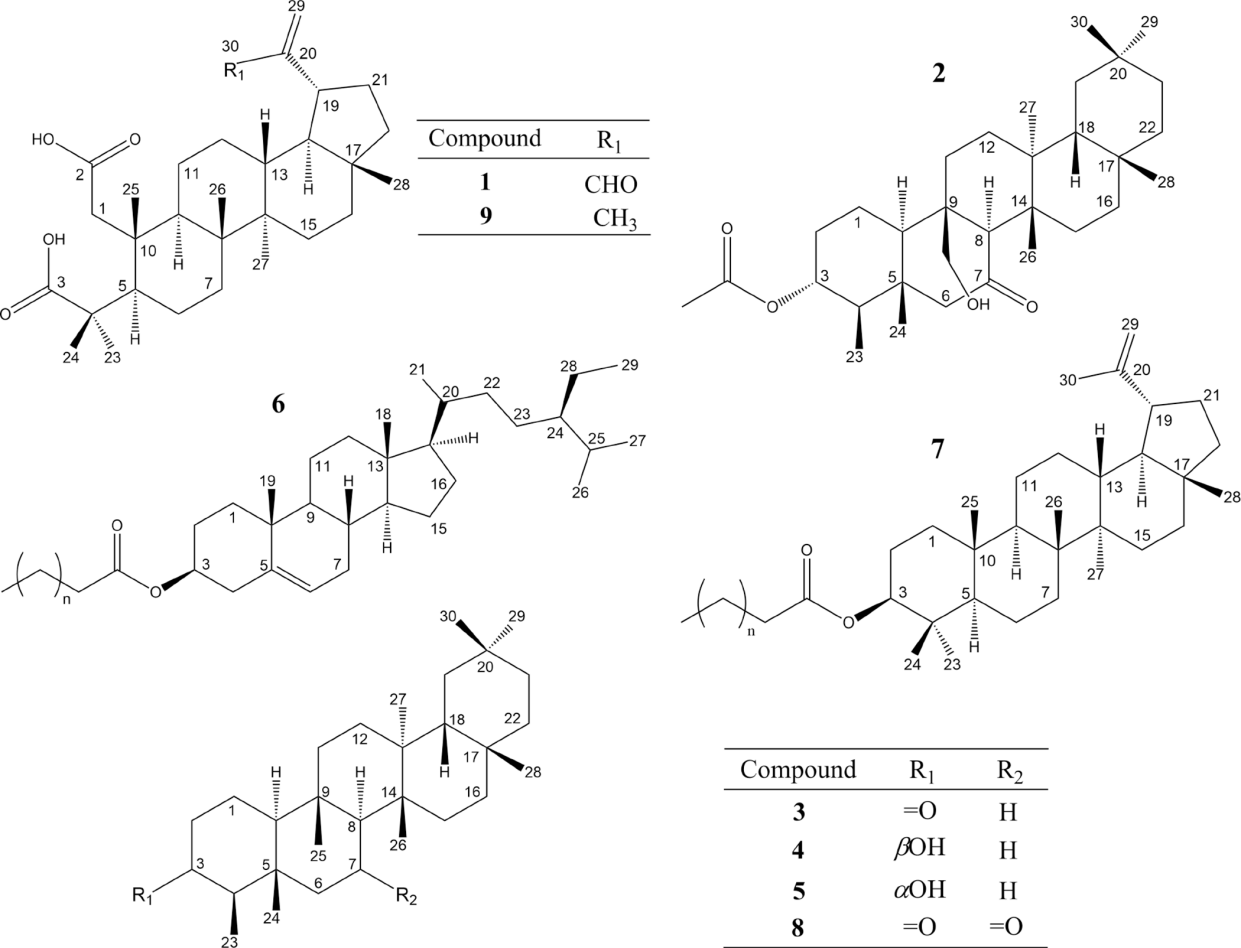
Compounds isolated from *Maytenus quadrangulata* branches.

## Results and Discussion

Compound **1** was obtained as a slightly yellowish white powder, [α]_*D*_^25^ = +6.67°(*c* 0.3, CHCl_3_). The molecular formula C_30_H_46_O_5_ was confirmed by HR-ESIMS with *m*/*z* 509.32486 [M + Na]^+^ (*m*/*z* calc. 509.3237). The IR spectrum exhibited a broad band from 2500 to 3500 cm^–1^, a band at 1461.1 cm^–1^ characteristic of an out-of-plane bending vibration of the C–H bond in a terminal alkene, a band at 1261.64 cm^–1^ and a sharp band at 1695.10 cm^–1^, characteristics of carbonyl groups. The ^1^H NMR spectrum presented five singlets (δ_*H*_ 0.81, 0.92 (6H), 1.01, 1.18, and 1.24) corresponding to six methyl groups and two doublets at δ_*H*_ 2.44 (*J* = 19.2 Hz) and δ_*H*_ 2.62 (*J* = 19.2 Hz), characteristic of hydrogens adjacent to a carbonyl group. Moreover, there were two singlets at δ_*H*_ 6.29 (1H) and δ_*H*_ 5.93 (1H), designated for alkene hydrogen atoms, and one singlet at δ_*H*_ 9.51 (1H) indicating the presence of an aldehyde (Table 1). On the other hand, the ^13^C NMR spectrum exhibited two signals at δ_*C*_ 187.0 and δ_*C*_ 178.0, and one signal at δ_*C*_ 195.3, characteristics of carbonyl carbons from carboxylic acid and aldehyde, respectively. Furthermore, two additional signals at δ_*C*_ 166.7 and 133.6 indicate the presence of *sp* ^2^-hybridized carbons. In light of this, the ^13^C NMR spectral data were compared to 2,3-*seco*-lup-20­(29)-ene-2,3-dioic acid (**9**)^12^ and 3β-hydroxy-lupan-20(29)-en-30-al,^12^ suggesting that compound **1** displays a lupane skeleton with an aldehyde group at C-30. The HSQC spectrum revealed a correlation between C-30 (δ_*C*_ 195.3) and the singlet at δ_*H*_ 9.51, as well as between C-29 (δ_*C*_ 133.6) and δ_*H*_ 6.29 and δ_*H*_ 5.93, signals characteristic of an exomethylene group typical of lupene-type triterpenes. The HMBC spectrum showed correlations between C-30 and H-29, confirming the presence of an aldehyde group at carbon C-30. The HSQC spectrum displayed a correlation between C-1 (δ_*C*_ 41.1) and the doublets at δ_*H*_ 2.44 and δ_*H*_ 2.62, which, in the HMBC spectrum, were correlated with the signal at δ_*C*_ 178.0 (C-2). Additionally, the HSQC spectrum exhibited a correlation between the signals at δ_*C*_ 20.5 (C-25) and δ_*H*_ 0.92 (H-25). In the HMBC spectrum, the latter was also correlated with C-1 (δ_*C*_ 41.1), C-2 (δ_*C*_ 178.0), C-5 (δ_*C*_ 48.3), C-9 (δ_*C*_ 41.8), and C-10 (δ_*C*_ 40.7). These correlations support the presence of a carboxylic acid group at C-2. In the HSQC spectrum, the methyl groups C-23 (δ_*C*_ 28.8) and C-24 (δ_*C*_ 22.1) were correlated with the signals at δ_*H*_ 1.24 (H-23) and at δ_*H*_ 1.18 (H-24), respectively. The HMBC spectrum revealed correlations between H-23 and H-24 with C-3 (δ_*C*_ 187.3), C-4 (δ_*C*_ 45.9), and C-5 (δ_*C*_ 48.3), confirming the presence of a carboxylic acid at C-3. In the NOESY spectrum, correlations were observed between H-25 (δ_*H*_ 0.92) and H-26 (δ_*H*_1.01), H-26 and H-13β (δ_*H*_1.64), as well as between H-28 (δ_*H*_0.81) and H-13β and H-15β (δ_*H*_1.68). Additionally, correlations between H-9α (δ_*H*_2.42) and H-27 (δ_*H*_0.92), and between H-27 and H-18α (δ_*H*_2.49), were detected. These correlations are consistent with the proposed relative stereochemistry of compound 1. Furthermore, the well-established stereochemistry of the carbons was assumed here because all lupane triterpenes are formed through the same mechanism, sharing the same absolute configuration.^13^ The biosynthesis of 2,3-*seco*-triterpenoids appears to originate from a postcyclization mechanism, as 2,3-*seco*-lupanes retain the C-20–C-29 unsaturation. Based on this, it is proposed that the biosynthesis of 30-oxo-2,3-*seco*-lup-20­(29)-ene-2,3-dioic acid (compound **1**) begins with the polycyclization of oxidosqualene catalyzed by an oxidosqualene cyclase, yielding lupeol. The opening of ring A to form the 2,3-*seco* structure occurs via an oxidative sequence involving the formation of a diketone, followed by a Baeyer–Villiger rearrangement and hydrolysis, resulting in carboxylic acids at C-2 and C-3. The 2,3-*seco* triterpene (compound **9**) may also be oxidized at C-30 by a monooxygenase, either before or after ring A opening (Figure 2). 2,3-*seco*-Triterpenoids constitute the second most reported class of *seco*-triterpenoids, although their occurrence is restricted to a few plant families.^14,15^ Therefore, compound **1** was identified as a new lupane, 30-oxo-2,3-*seco*-lup-20­(29)-ene-2,3-dioic acid, based on the NMR data (Table 1).

**Figure 2:**
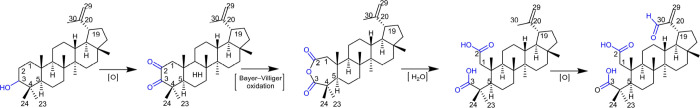
Proposed biosynthetic pathway for *seco*-triterpenoids.

**Table 1:** NMR Spectral Data of Compound **1** and Comparison with ^13^C NMR Data of 2,3-*seco*-lup-20­(29)-ene-2,3-dioic Acid^12^ (a) and 3β-hydroxy-lupan-20(29)-en-30-al^12^ (b)

atom	type	δ_*C*_ ^a^	δ_*H*_	HMBC	COSY	NOESY	δ_*C*_ ^a^ (a)^12^	δ_*C*_ ^a^ (b)^12^
1	CH_2_	41.1	2.44; d *J* 19.2	2	1		40.9	38.70
			2.62; d *J* 19.2					
2	C	178.0					178.5	27.40
3	C	187.0					187.5	78.99
4	C	45.9					45.6	38.87
5	CH	48.3	2.49			7α	48.2	55.29
6	CH_2_	21.5	1.29	5			21.3	18.30
			1.29					
7	CH_2_	33.8	1.53β		7		33.7	34.28
			2.34α					
8	C	41.9					41.8	40.77
9	CH	41.8	2.42				41.7	50.24
10	C	40.7					40.7	37.14
11	CH_2_	21.5	1.29		9		19.2	20.94
12	CH_2_	22.7	1.29				24.9	27.64
			1.29					
13	CH	37.6	1.64			26	37.9	37.73
14	C	43.1					43.2	42.69
15	CH_2_	27.3	1.02α	14,17	15,16		27.5	27.34
			1.68β					
16	CH_2_	35.4	1.45		15		35.5	35.39
			1.53					
17	C	43.3					43.2	43.28
18	CH	48.3	2.49	19			48.4	51.2
19	CH	48.3	2.49	18	21		48.0	36.70
20	C	166.7					150.9	157.0
21	CH_2_	33.7	1.53				29.8	32.60
			1.53					
22	CH_2_	39.9	1.41				39.9	39.93
			1.41					
23	CH_3_	28.8	1.24; s	24,3,5,4			29.8	27.99
24	CH_3_	22.1	1.18; s	23,4,5,3			21.3	15.37
25	CH_3_	20.5	0.92; s	10,1,9,5,2		26	20.8	16.07
26	CH_3_	15.9	1.01; s	7,9		15β,7β	15.9	15.94
27	CH_3_	14.4	0.92; s	15,13		15α,18,9	14.6	14.41
28	CH_3_	17.8	0.81; s	16,22,17		15β,13	18.0	17.79
29	CH_2_	133.6	5.93; s	30			109.4	132.90
			6.29; s					
30	CH	195.3	9.51; s				40.9	195.08

a CDCl_3_.

In the phytochemical study of the chloroform extract of *M. quadrangulata* branches, mixtures of hydroxylated triterpenes were identified. The direct separation of these compounds proved difficult, even after the acetylation reaction. Nevertheless, it was possible to determine the structure of the major compound, identified as a semisynthetic acetylated triterpenoid derivative, whose structure was proposed based on NMR data (Table 2). The ^1^H NMR spectrum showed six singlets (δ_*H*_ 0.86; 0.95; 0.99; 1.03; 1.17 and 1.40) relating to six methyl groups and one doublet at δ_*H*_ 0.76 (3H; *J* = 6.7 Hz), characteristic of H-23 in a friedelane skeleton. A signal at δ_*H*_ 3.49 (2H) is assigned to hydrogens bonded to a hydroxylated carbon. Two doublets at δ_*H*_ 2.26 (1H, *J* = 11.9 Hz) and δ_*H*_ 2.17 (1H, *J* = 11.9 Hz), along with a singlet at δ_*H*_ 2.74 (1H), indicate the presence of hydrogens adjacent to a carbonyl group. A triplet of doublet at δ_*H*_ 4.62 (1H; *J* = 11.1;5.1 Hz) is characteristic of hydrogens adjacent to an acetoxy group, and a signal at δ_*H*_ 2.04 (3H) corresponds to the methyl hydrogens of this group. The ^13^C NMR spectrum showed signals at δ_*C*_ 211.5 and δ_*C*_ 170.8, characteristic of the carbonyl carbons of a ketone and an ester, respectively. There were also signals at δ_*C*_ 65.9 and δ_*C*_ 74.3, indicating the presence of a hydroxymethyl and an acetoxy group, in that order. That being so, a literature review was conducted, revealing that the signals were close to those of 25-hydroxyfriedelan-3-one^16^ and 3α-hydroxyfriedelan-7-one.^9^ Thus, the NMR data of these compounds were compared to the most intense peaks in the ^13^C NMR spectrum of the sample, suggesting the presence of a friedelane-type triterpene with a carbonyl group at C-7 (δ_*C*_ 211.5), a hydroxyl group at C-25 (δ_*C*_ 65.9) and an acetoxy group at C-3 (δ_*C*_ 74.3) (Table 2).

**Table 2:** NMR Spectral Data of Compound **2** and Comparison with ^13^C NMR Data of 3*α*-hydroxyfriedelan-7-one^9^ (a) and 25-hydroxyfriedelan-3-one^16^ (b)

atom	type	δ_*C*_ ^a^	δ_*H*_	HMBC	COSY	δ_*C*_ ^a^ (a)^9^	δ_*C*_ ^a^ (b)^16^
1	CH_2_	19.6	1.74			19.7	24.7
			1.46				
2	CH_2_	36.4	1.5			36.2	42.6
3	CH	74.3	4.62; td; *J* 11.1; 5.1		4	70.8	212.9
4	CH	49.9	1.57	3	3	53.1	58.5
5	C	44.4	-			44.2	42.4
6	CH_2_	57.6	2.26; d; *J* 11.9	7		57.8	41.8
			2.17; d; *J* 11.9				
7	C	211.5	-			212.1	17.9
8	CH	63.7	2.74	7,9,14,26		63.4	53.7
9	C	42.7	-			42.7	42.0
10	CH	59.7	1.57	11		59.8	60.7
11	CH_2_	35.1	1.2	12,25	12	36.3	29.9
12	CH_2_	32.3	1.06	14	11	29.9	31.2
13	C	39.5	-			39.3	39.7
14	C	37.6	-			37.4	37.7
15	CH_2_	32.2	1.27	26		31.9	32.7
16	CH_2_	35.9	1.39			35.5	36.0
17	C	30.1	-			30.1	30.1
18	CH	41.9	1.56			41.8	42.7
19	CH_2_	35.7	1.37			34.9	35.3
20	C	28.2	-			28.0	28.1
21	CH_2_	32.9	1.5			32.8	32.7
			1.2				
22	CH_2_	38.8	1.5			38.6	39.2
			0.94				
23	CH_3_	10.1	0.76; d; *J* 6.7	5,3,4		10.1	7.0
24	CH_3_	14.9	0.86; s	10,6,5,4		14.7	14.7
25	CH_2_	65.9	3.49; s			19.3	63.0
26	CH_3_	19.5	1.40; s	8,13,14,15		19.5	20.1
27	CH_3_	18.4	1.03; s	12,13,14,18		18.2	18.5
28	CH_3_	31.9	1.17; s	18,22,16,17		31.6	32.1
29	CH_3_	34.7	0.95; s	19,20,21,30		34.5	35.0
30	CH_3_	31.7	0.99; s	19,20,21,29		32.1	31.7
1′	C	170.8					
2′	CH_3_	21.3	2.04	1’			

a CDCl_3_.

The HSQC spectrum displayed correlations between the triplet of doublet at δ_*H*_ 4.62 and the signal at δ_*C*_ 74.3 (C-3), and between the doublet at δ_*H*_ 0.76 (3H, *J* = 6.7 Hz) and C-23 at δ_*C*_ 10.1. The HMBC spectrum correlated C-3 with H-23 (δ_*H*_ 0.76), and C-1′ (δ_*c*_ 170.8) with H-2′ (δ_*H*_ 2.04). The chemical shift of H-3 here (δ_*H*_ 4.62) agrees with the values reported for compounds containing an acetoxy group at C-3. Furthermore, in the HSQC spectrum C-8 (δ_*C*_ 63.7) is correlated with δ_*H*_ 2.74; and C-6 (δ_*C*_ 57.6) with the signals at δ_*H*_ 2.26 (1H, *J* = 11.9 Hz) and δ_*H*_ 2.17 (1H, *J* = 11.9 Hz). The HMBC spectrum presented correlations of C-7 (δ_*C*_ 211.5) with H-8 (δ_*H*_ 2.74) and H-6 (δ_*H*_ 2.18 and 2.27), confirming the presence of a carbonyl group at C-7. On the other hand, the HMBC spectrum displayed correlations of H-11 (δ_*H*_ 1.2) with C-25 and C-10 (δ_*C*_ 59.7); of C-10 with H-24 (δ_*H*_ 0.86), and of H-8 (δ_*H*_ 2.74) with C-9 (δ_*C*_ 42.7) and C-14 (δ_*C*_ 37.6). Ultimately, in the HSQC spectrum, the signal at δ_*H*_ 3.49 was correlated with C-25 (δ_*C*_ 65.9), whose values are closely related to those of 25-hydroxyfriedelan-3-one,^16^ suggesting the presence of a hydroxyl group at C-25. The stereochemistry at C-3 was established from the multiplicity and coupling constants of H-3. The H-3 signal appears as a triplet of doublets at δ_*H*_ 4.62 (*J* = 11.1 and 5.1 Hz), showing a large axial–axial coupling (11.1 Hz) with H-4 and H-2α and a smaller axial–equatorial coupling (5.1 Hz) with H-2β. These data confirm an α-acetoxy group at C-3 and H-3 in the β position. The absolute stereochemistry of the remaining carbons was assumed based on biosynthetic considerations, as friedelane triterpenes are formed via the same pathway and share a common absolute configuration. Therefore, compound **2** was identified as a new semisynthetic friedelane, 25-hydroxy-7-oxofriedelan-3α-yl acetate, and it is noteworthy that its hydroxylated natural product precursor (7-oxofriedelane-3α,25-diol) has never been reported in the literature, making it also a novel molecule.

The chemical structure of the known compounds **3**–**9** was determined by comparing their ^13^C NMR signals and the data available in the literature (Tables S1–S3). They were identified as friedelan-3-one (**3**),^17^ friedelan-3β-ol (**4**),^18^ friedelan-3α-ol (**5**),^18^ a mixture of β-sitosterol alkanoate (**6**)^19^ and lupeol alkanoate (**7**),^20^ friedelane-3,7-dione (**8**)^21^ and 2,3-*seco*-lup-20­(29)-ene-2,3-dioic acid (**9**).^12^ Compounds **6**, **7**, and **9** are reported for the first time as metabolites of *M. quadrangulata* in this work. The family Celastraceae is known to be rich in pentacyclic triterpenes. Friedelane-type triterpenes, particularly friedelin and β-friedelinol, are frequently reported in species of this family, especially within the genus *Maytenus*.^12^

### Biological Activity

Previous studies have reported that friedelan-3-one (**3**), commonly isolated from species of the Celastraceae family, exhibits several biological activities, including antiviral, hepatoprotective, anti-inflammatory, antimicrobial, and analgesic.^22–25^ Friedelan-3β-ol (**4**) exhibits antimicrobial and antiangiogenic properties and is frequently found in species of the *Maytenus* genus.^26,27^ Friedelan-3α-ol (**5**) has shown cytotoxic activity against colorectal cancer (Caco-2) and human hepatocarcinoma (HepG2) cell lines, as well as anti-inflammatory activity.^28,29^ Friedelane-3,7-dione (**8**) inhibits the enzyme acetylcholinesterase and has demonstrated cytotoxic activity against three human cancer cell lines: HL-60 (acute leukemia), BEL-7402 (hepatocellular carcinoma), and A549 (lung adenocarcinoma).^30,31^ In addition, the activity of 2,3-*seco*-lup-20­(29)-ene-2,3-dioic acid (**9**) has also been reported in the literature. Compound **9** exhibited potent cytotoxicity against 786-O (kidney) and HT-29 (colon) cancer cell lines, strong inhibitory activity against α-glucosidase, and moderate inhibition of HIV-1 protease.^32–34^

#### Cytotoxic Activity

The cytotoxic activity of compounds **1** and **9** was evaluated for the first time against the human acute monocytic leukemia cell line (THP-1) and the chronic myeloid leukemia cell line (K-562), using cytarabine and imatinib as positive controls, respectively (Table 3). Compounds **3**, **4**, **5**, and **8** were not tested in this work, because they have already been evaluated against THP-1 and K-562 cell lines.^9,35^ The biological assay with THP-1 was also conducted with lupeol, and the results are provided to enable direct comparison. Both compounds **1** and **9** exhibited cytotoxic activity, with IC_50_ values lower than that observed for the respective controls. Compound **1** was the most active, with IC_50_ values of 2.45 ± 0.41 μM against THP-1, and 18.16 ± 1.56 μM against K-562, suggesting that the carbonyl group at C-30 may play a significant role in its antileukemic activity. Nonetheless, compound **9** also demonstrated cytotoxic effects with IC_50_ = 6.27 ± 0.19 μM for THP-1 and IC_50_ = 34.90 ± 1.52 μM for K-562. Although the cytotoxic activity of compound **9** against kidney and colon cancer cell lines has been previously reported in the literature^32^ (GI_50_ of 0.5 and 2.9 μM, respectively), this is the first study with leukemia cell lines.

**Table 3:** Cytotoxicity of Compounds **1** and **9** against Leukemia Cell Lines^a^

	IC_50_ (μM) ± SD^b^	
sample	THP-1	K-562
Compound **1**	2.45 ± 0.41	18.16 ± 1.56
Compound **9**	6.27 ± 0.19	34.90 ± 1.52
lupeol	155.90 ± 10.12	NT
cytarabine	40.75 ± 4.45	NT
imatinib	NT	41.57 ± 33.00

a THP-1: human acute monocytic leukemia; K-562: chronic myeloid leukemia; NT: Not tested.

b Values presented as mean ± standard deviation (SD).

Lupeol is a lupane-type triterpene with a well-documented broad range of biological activities, such as anticancer, anti-inflammatory, and antimicrobial. The anticancer activity of lupeol, evidenced in models of bladder, lung, liver, and colorectal cancers, is mediated by diverse mechanisms, including inducing apoptosis, inhibiting migration and invasion of cancer cells, and suppressing cell proliferation, suggesting utility in both treatment and prevention.^36^ Nonetheless, despite its multifaceted anticancer effects, lupeol exhibited significantly weaker activity against THP-1 cells (IC_50_ = 155.90 ± 10.12 μM) compared to compounds **1** and **9**.

Previous work revealed that the lupeol oxidation at C-30 leading to α,β-unsaturated aldehyde increases cytotoxicity against human T-cell acute lymphoblastic leukemia cell line (JURKAT) and K-562 leukemic cells.^37^ Indeed, several studies have demonstrated that incorporating this group results in derivatives with enhanced antileukemic activity compared to nonfunctionalized analogues.^38,39^ Noh-Burgos et al. evaluated the cytotoxic activity of semisynthetic triterpenes with a lupane skeleton, and among all the compounds and cell lines tested, the best activities were observed for those containing an α,β-unsaturated aldehyde group at C-30.^40^ A proposed mechanism of action involves the formation of Michael-type adducts from the reaction with cellular thiols, which are frequently overexpressed in tumor cells. A notable feature is its low reactivity toward hydroxyl and amino groups found in nucleic acids, thereby avoiding carcinogenic and mutagenic side effects. Moreover, in addition to reacting with cellular constituents, this type of compound can also trigger apoptosis via the mitochondrial pathway, an alternative mechanism of cell death.^41^ All these findings underscore the role of the aldehyde group at C-30 in contributing to the elevated cytotoxicity observed for compound **1**.

Furthermore, because the triterpene lacking the aldehyde group at C-30 demonstrated superior efficacy relative to lupeol, the scaffold’s open-ring may also influence the activity. Previous studies have shown that 30-hydroxy-2,3-*seco*-lup-20­(29)-ene-2,3-dioic acid, a molecule structurally very similar to compounds **1** and **9**, exhibits significant anti-inflammatory activity, demonstrating the strongest effects on superoxide anion generation and elastase release among the 11 molecules tested, with IC_50_ values of 0.06 ± 0.01 and 1.03 ± 0.35 μg/mL, respectively.^42^ The cleavage of a C–C bond increases the flexibility and, consequently, enhances the interaction with a biological target, which may account for the higher cytotoxicity observed for compounds **1** and **9**. In fact, structural modifications that break the ring A producing A-*seco*-triterpenoids have been a promising approach to increasing biological efficacy.^43,44^ Additionally, the presence of two polar carboxylic acid groups may increase affinity toward target molecules. To confirm this, however, a complete structure–activity relationship analysis is necessary.

On the other hand, given that the cell line K-562 exhibits broad resistance to apoptosis triggered by multiple chemotherapeutic agents, such as etoposide and cisplatin, the discovery of novel cytotoxic compounds that enhance apoptotic susceptibility is essential for advancing more effective therapeutic strategies.^45^ A study showed that two of the most common lupane triterpenes, betulin and betulinic acid, displayed cytotoxicity against the K-562 cell line, with IC_50_ values of 12.89 ± 1.44 and 21.96 ± 1.15 μM, respectively.^46^ This work further demonstrated that their derivatives containing a triphenylphosphonium cation moiety exhibited a marked increase in cytotoxicity, with IC_50_ values reaching 0.57 ± 0.03 μM. This is attributed to their greater bioavailability and aqueous solubility, which leads to a higher intracellular accumulation. Therefore, although the IC_50_ value of compound **1** was higher than that of betulin, its cytotoxic potential may be improved through structural modifications that enhance its bioavailability.

Pereira et al.^47^ reported the cytotoxicity of various triterpenes against THP-1 and K-562 cells, identifying α-amyrin as the most potent with IC_50_ of 9.92 ± 0.82 and 8.45 ± 0.85 μM, respectively. Finally, in a study conducted by Oliveira et al.,^48^ all evaluated compounds exhibited IC_50_ values exceeding 259 μM in the cell viability assay using THP-1 and K-562 cells. Hence, these findings demonstrate the strong efficacy of compound **1** in inhibiting leukemia cell proliferation, particularly THP-1, when compared with other triterpenes and their derivatives reported in the literature.

Ultimately, these results highlight the potential of compounds **1** and **9** as anticancer agents, and additional studies should be conducted to assess their activity against other cancer cell lines. Furthermore, further in-depth investigations are warranted to elucidate their mechanisms of action.

### Drug-Likeness and ADMET Properties

Given the promising outcomes from the biological assays for compounds **1** and **9**, an *in silico* ADMET analysis was performed using ADMETLab 3.0 to obtain a preliminary assessment of their pharmacokinetic and toxicity profile. The predicted parameters are summarized in Table 4 along with their compliance with general drug-likeness rules.

**Table 4:** Selected *In Silico* ADMET Parameters for Compounds **1**, **9**, and lupeol Using ADMETLab 3.0

*Physicochemical and structural properties*				
**Descriptors**	**1**	**9**	**Lupeol**	**Optimal range** ^a^
MW (g/mol)	486.33	472.36	426.39	100–600
logP (log mol/L)	4.702	5.232	6.729	0–3
logS (log mol/L)	–4.317	–4.496	–7.205	–4–0.5
HBD	2	2	1	≤7
HBA	5	4	1	≤12
nRtB	6	5	1	≤11
TPSA (Å^2^)	91.67	74.6	20.23	≤180
Fsp^3^	0.833	0.867	0.933	≥0.42

*Absorption*				
**Descriptors**	**1**	**9**	**Lupeol**	**Optimal range** ^a^
Caco-2 (log cm/s)	–5.383	–5.337	–5.208	>−5.150
MDCK	–4.996	–5.069	–4.983	>−4.699
HIA	0.003	0	0	0–0.3

*Distribution*				
**Descriptors**	**1**	**9**	**Lupeol**	**Optimal range** ^a^
PPB (%)	97.50	97.03	97.58	≤90

*Elimination*				
**Descriptors**	**1**	**9**	**Lupeol**	**Optimal range** ^a^
*T*_1/2_ (h)	1.299	1.264	0.248	≥8
CL_*plasma*_	0.840	0.909	11.739	0–5

*Toxicity*				
**Descriptors**	**1**	**9**	**Lupeol**	**Optimal range** ^a^
ROAT (mL/min/kg)	0.226	0.208	0.389	0–0.3

*Drug-likeness rules*				
**Rule**	**1**	**9**	**Lupeol**	**Requirements** ^a^
Lipinski	yes	yes	yes	MW ≤ 500, logP ≤ 5, HBA ≤ 10, HBD ≤ 5
Pfizer	no	no	no	logP < 3, TPSA > 75
GSK	no	no	no	MW ≤ 400, logP ≤ 4
Veber	yes	yes	yes	TPSA ≤ 140, nRtB ≤ 10

a Optimal ranges correspond to the reference values suggested by ADMETLab 3.0 and are provided for comparative purposes only. **Abbreviations:** MW, molecular weight (g/mol), logP, n-octanol/water partition coefficient (log mol/L); logS, aqueous solubility (log mol/L); HBD, hydrogen bond donors; HBA, hydrogen bond acceptors; nRtB, number of rotatable bonds; TPSA, topological polar surface area (Å^2^); Fsp^3^, fraction of sp^3^-hybridized carbons; Caco-2, human colon adenocarcinoma cell permeability (log cm/s); MDCK, Madin–Darby canine kidney cell permeability; HIA, human intestinal absorption (expressed as a probability (0–1)); PPB, plasma protein binding (%); *T*_1/2_, half-life (h); CL_*plasma*_, plasma clearance (mL/min/kg); ROAT, rate of oral acute toxicity (expressed as a probability (0–1)).

All molecules satisfy Lipinski’s and Veber’s rules, suggesting acceptable oral drug-likeness in terms of molecular size, polarity, and molecular flexibility. Compound **1**, however, is the only one that does not violate the lipophilicity requirement (logP ≤ 5), whereas compound **9** and lupeol exceed this threshold, reflecting their highly lipophilic triterpenoid frameworks. Conversely, none of the three metabolites fulfills the criteria established by GSK’s and Pfizer’s rules, mainly due to elevated logP values and molecular weight, which is a known limitation of pentacyclic triterpenes and related derivatives. The low predicted aqueous solubility, indicated by logS < −4, was expected for all compounds due to their long saturated carbon chains, as evidenced by high Fsp^3^ values. Nevertheless, the presence of ionizable groups in compounds **1** and **9** suggests that solubility may be improved through salt formation or formulation strategies, such as encapsulation in polymeric carriers or interaction with carrier proteins, approaches commonly applied to poorly soluble drug candidates.^49,50^ This interaction can be further investigated by molecular docking and dynamics.

Regarding absorption-related descriptors, both compounds present a topological polar surface area (TPSA) and a number of rotatable bonds within the recommended ranges according to Veber’s rule. These parameters indicate the molecular flexibility and its ability to penetrate the cell membrane. Compound **1** exhibits the highest TPSA, primarily due to the carboxyl and aldehyde groups, which may contribute to improved polar interactions relative to lupeol. In addition, as discussed previously, the greatest nRtB values found for compounds **1** and **9** compared to lupeol are expected, given that they are *seco*-triterpenoids. The predicted Caco-2 (human colon adenocarcinoma cell permeability) and MDCK (Madin–Darby canine kidney cell permeability) permeability values for the compounds suggest low to moderate permeability; however, the Human Intestinal Absorption (HIA) probabilities indicate favorable oral absorption.

Distribution parameters reveal high plasma protein binding (PPB > 97%) for all compounds, which may limit their free circulating fraction and reduce their effective concentration at the target site, yet is commonly observed for lipophilic triterpenes. The excretion descriptors reveal the body’s overall capacity to eliminate the drug. Both molecules isolated in this work exhibit low plasma clearance and short half-life, suggesting rapid elimination and limited tissue distribution. The predicted acute toxicity in rats is an important parameter for assessing the safety of potential drug candidates; hence, the low ROAT values of compounds **1** and **9** suggest their potential safety at preliminary stages. In summary, the *in silico* ADMET analysis revealed a mixture of favorable and unfavorable outcomes across the different descriptors. Nevertheless, one must consider the methodological limitations inherent to the predictive approach used here. Although these predictions are inherently limited and do not replace experimental pharmacokinetic studies, they provide a useful comparative framework. Within this context, the novel compound (**1**) displayed the most balanced profile among the evaluated molecules, and lupeol showed the least advantageous values compared with compounds **1** and **9**, underscoring the therapeutic potential of the triterpenes isolated in this work as lead structures for further optimization.

## Conclusion

The phytochemical study of the chloroform extract of *Maytenus quadrangulata* branches led to the isolation of the new triterpene 30-oxo-2,3-*seco*-lup-20­(29)-ene-2,3-dioic acid (**1**) and the new semisynthetic triterpene 25-hydroxy-7-oxofriedelan-3α-yl acetate (**2**), as well as seven other known compounds: friedelan-3-one (**3**), friedelan-3β-ol (**4**), friedelan-3α-ol (**5**), a mixture of long-chain fatty acids of β-sitosterol and lupeol—β-sitosterol alkanoate (**6**) and lupeol alkanoate (**7**)-, friedelan-3,7-dione (**8**) and 2,3-*seco*-lup-20­(29)-ene-2,3-dioic acid (**9**). Compounds **6**, **7** and **9** are reported in *M. quadrangulata* for the first time. Compound **1** exhibited the highest cytotoxicity against the THP-1 and K-562 cell lines and the most favorable drug-like profile, highlighting its potential as a candidate anticancer therapy agent.

## Experimental Section

### General Experimental Procedures

The chromatographic columns (CC), thin-layer chromatography (TLC), and preparative thin-layer chromatography (PTLC) plates were prepared using silica gel 60 (70–230 mesh or 230–400 mesh), silica gel 60 G (0.25 mm), and silica gel 60 G (1 mm) with a fluorescence indicator at 254 nm, respectively. For TLC, a solution (1:1 v/v) of vanillin in ethanol (1:99 v/v) and perchloric acid in water (3:97 v/v) was used as a developing reagent, followed by heating to 100 °C. 1D and 2D NMR spectra were obtained on Bruker Avance DRX-600 or DRX-400 spectrometers, with CDCl_3_ as solvent. Chemical shifts (δ) were recorded in ppm using tetramethylsilane (TMS; δ_*H*_ = δ_*C*_ = 0) as an internal reference standard, and coupling constants (*J*) were expressed in Hz. Mass analyses were performed on a Q-Exactive high-resolution mass spectrometer (Thermo Scientific) equipped with a heated electrospray ionization source (H-ESI), operating in positive ion mode, using direct infusion. The infrared spectrum was recorded using a Shimadzu IRSpirit-T spectrometer equipped with an attenuated total reflection (ATR) accessory.

### Plant Material

The branches of *Maytenus quadrangulata* were collected in the district of Brejo do Amparo, municipality of Januária, Minas Gerais, Brazil, in December 2019. The identification and collection of the plant material were carried out by Professor Dr. Maria Olívia Mercadante Simões from the Department of General Biology at the State University of Montes Claros. A voucher specimen (HMC, Number 405) was deposited in the Herbarium of the Universidade Estadual de Montes Claros. The botanical material was registered with Conselho de Gestão do Patrimônio Genético (CGEN/SisGen), Brazil, under the registration number A639A9E.

### Extraction and Isolation

The branches of *M. quadrangulata* were dried at room temperature and subsequently ground. The powdered material (2.1 kg) was subjected to extraction by sequential maceration with hexane and chloroform. After filtration, the solvents were removed using a rotary evaporator at reduced pressure to afford the respective extracts. Upon adding 100 mL of acetone to the chloroform extract (12.4 g), a solid precipitated and was filtered under reduced pressure, yielding the solid (**SCE**: 3.4 g) and the filtrate (**FCE**: 9.0 g).

**SCE** was purified by CC using silica gel 60 (230–400 mesh) and hexane, chloroform, ethyl acetate, and methanol as eluents, either neat or in mixtures, in order of increasing polarity. A total of 205 fractions were obtained and grouped into 8 groups according to their TLC profiles (A1–A8). The A5 group (A5; fractions 110–155; chloroform/ethyl acetate(9:1); 24.0 mg) was subjected to other purification by CC using silica gel 60 (230–400 mesh), resulting in the following constituents: friedelan-3-one (**3**) (A5.1; hexane/ethyl acetate (9:1), 4.1 mg); friedelan-3β-ol (**4**) (A5.2; hexane/ethyl acetate (8.5:1.5), 3.2 mg); friedelan-3α-ol (**5**) (A5.3; hexane/ethyl acetate (7:3), 10.0 mg).

The filtrate, **FCE**, was purified by CC using silica gel 60 (230–400 mesh) and hexane, chloroform, ethyl acetate, and methanol as eluents, either neat or in mixtures, following an increasing polarity gradient. Thirty-five fractions were collected and grouped into 29 groups according to their TLC profiles (B1–B29). Group B2 (fractions 5–6; hexane/chloroform (1:1); 23.6 mg) underwent successive PTLC, leading to a mixture of β-sitosterol alkanoate (**6**) and lupeol alkanoate (**7**). Group B3 (fraction 12; chloroform; 27.3 mg) was identified as friedelan-3-one (**3**) and group B4 (fraction 13; chloroform; 31.2 mg) as a mixture of friedelan-3-one (**3**) and friedelan-3β-ol (**4**). Group B5 (fraction 16; chloroform/ethyl acetate (9:1); 592.2 mg) was subjected to successive CC (silica gel 60, 230–400 mesh) and PTLC, resulting in two subfractions identified as friedelan-3α-ol (**5**) (7.5 mg) and friedelane-3,7-dione (**8**) (2.6 mg). Group B6 (fractions 19; chloroform/ethyl acetate (7:3); 1.22 g) was subjected to CC using Sephadex LH-20 as the stationary phase, and hexane/chloroform/methanol (2:1:1) as eluent. Eighteen fractions were obtained and grouped into three subgroups. B6.1 (fractions 3–18; 603.0 mg) was subjected to successive CC, yielding a mixture of unidentified triterpenes. Due to the challenging separation of the compounds of this fraction by conventional chromatography, an acetylation reaction was performed to facilitate their purification: B6.1.2 (7.6 mg), 2 mL of acetic anhydride, and 2 mL of pyridine were added to a 50 mL flask, and the mixture was kept under magnetic stirring for 20 h. At the end of the reaction, 20 mL of distilled water was added to the flask, and then a liquid–liquid extraction was performed with three 20 mL portions of chloroform, yielding 6.8 mg (B6.1.2.R) after solvent evaporation. Solid B6.1.2.R was subjected to PTLC using hexane/dichloromethane/ethyl acetate (48:48:4) as eluent, yielding a mixture containing 25-hydroxy-7-oxofriedelan-3α-yl acetate (**2**) (2.0 mg) as the major constituent. Group B7 (fraction 20; chloroform/ethyl acetate (1:1); 386.8 mg) was subjected to successive CC using Sephadex LH-20 and silica gel (230–400 mesh) as the stationary phase, resulting in the following constituents: 2,3-*seco*-lup-20­(29)-ene-2,3-dioic acid (**9**) (chloroform/ethyl acetate (8:2); 31.9 mg) and 30-oxo-2,3-*seco*-lup-20­(29)-ene-2,3-dioic acid (**1**) (chloroform/ethyl acetate (7:3); 10.0 mg).

30-Oxo-2,3-*seco*-lup-20­(29)-ene-2,3-dioic acid (**1**). A slightly yellowish white powder; IR (ATR) ν/cm^–1^ 2851, 2922, 1695, 1261, 1166, 977; [α]_*D*_^25^ = +6.67°(*c* 0.3, CHCl_3_); HR-ESI-MS [M + Na]^+^ *m*/*z* at 509.32486 (calc. 509.3237). ^1^H and ^13^C NMR data, see Table 1.

### Biological Activity

The cytotoxicity of compounds **1** and **9** was evaluated against the human tumor cell lines THP-1 (acute monocytic leukemia, ATCC-TIB-202^TM^) and K-562 (chronic myeloid leukemia, ATCC–CCL-243), by the MTT [3-(4,5-dimethylthiazol-2-yl)-2,5-diphenyltetrazolium bromide] colorimetric assay. Following centrifugation (70 × g, 5 min), the cell pellet was resuspended in complete medium and seeded into a 96-well plate at a density of 1 × 10^5^ cells per well. The plate was then incubated for 24 h at 37 °C in a humidified atmosphere with 5% CO_2_. The positive controls, cytarabine for THP-1 and imatinib for K-562, and test samples were serially diluted in culture medium supplemented with 1% fetal bovine serum (FBS) to concentrations of 100, 10, 1, and 0.1 μg/mL and added to the plate. Following incubation for 48 h, 100 μL of MTT solution (0.5 mg/mL) was added per well, and the plate was incubated for another 3 h. The supernatant was removed, and the resulting formazan crystals were dissolved in 100 μL of DMSO. The absorbance was measured at 550 nm on a Versamax microplate reader (Molecular Devices). Cytotoxicity was determined based on the sample concentration required to inhibit 50% of cell viability (IC_50_) relative to cells cultured without the test compounds (negative control). Data were obtained from two independent experiments.

### Drug-Likeness and ADMET Properties

The absorption, distribution, metabolism, excretion, and toxicity (ADMET) of compounds **1** and **9** were evaluated in ADMETLab 3.0.^51,52^ Fifteen properties were determined: molecular weight (MW), logarithm of the n-octanol/water partition coefficient (logP), and of aqueous solubility (logS); number of hydrogen bond donors (HBD), hydrogen bond acceptors (HBA), and rotatable bonds (nRot); Topological Polar Surface Area (TPSA), fraction of sp^3^ hybridized carbon (Fsp^3^); Human Colon Adenocarcinoma (Caco-2) and Madin-Darby Canine Kidney (MDCK) cells permeability, both in logarithmic scale; Human Intestinal Absorption (HIA); Plasma Protein Binding (PPB); Plasma Clearance (CL_plasma_), Half-life (*T*_1/2_), and Rate Oral Acute Toxicity (ROAT). In addition, their drug-like profiles were analyzed considering three well-known empirical rules: Lipinski’s (MW ≤ 500 Da; logP ≤ 5; HBA ≤ 10; HBD ≤ 5),^53^ Pfizer’s (logP < 3; TPSA > 75 Å^2^),^54^ GSK’s (MW ≤ 400 and logP ≤ 4),^55^ and Veber’s (TPSA ≤ 140 Å^2^, nRotB ≤ 10).^56^ These rules do not allow any violation, except Lipinski’s, which allows one. For comparative purposes, the analysis was also conducted for lupeol. The SMILES were obtained using Marvin and are provided in the Supporting Information, together with the Radar Maps from the analysis.

## Supplementary Material



## Notes

NMR spectra of compounds **1**–**9**, infrared (IR) spectrum, HR-IT-TOF-MS analysis of compound **1** (Figures S1–S30), NMR data comparison for compounds **3**–**9** (Tables S1–S3) SMILES strings and the radar maps of compounds **1**, **9** and lupeol (Figures S31–S33); and unannotated (raw) NMR spectra of compounds **1** and **2** (PDF)
