# Supplementary material for: Lupane Triterpenes with Antileukemia Activity from Maytenus quadrangulata
Source: ACS Omega. 2026 Mar 27;11(13):20806–15. doi: 10.1021/acsomega.5c13073 (PMC13063062; doi:10.1021/acsomega.5c13073)
Supplement: Supplementary file 1 [file ao5c13073_si_001.pdf]

Supporting Information

Lupane Triterpenes with Antileukemia Activity from *Maytenus quadrangulata*

Sandy V. M. Quintão[a], Mariana G. de Aguilar[a], Lucas C. Souza[a], Túlio R. Freitas[b], Maria E. C. dos S. Jardim[b], Lohanne B. E. de Souza[b], Adriano de P. Sabino[b], Lucienir P. Duarte[a] and Grasiely F. de Sousa[a]

[a]Departamento de Química, Universidade Federal de Minas Gerais, 31270-901 Belo Horizonte-MG, Brasil

[b]Departamento de Análises Clínicas e Toxicológicas, Faculdade de Farmácia, Universidade Federal de Minas Gerais, 31270-901 Belo Horizonte-MG, Brasil

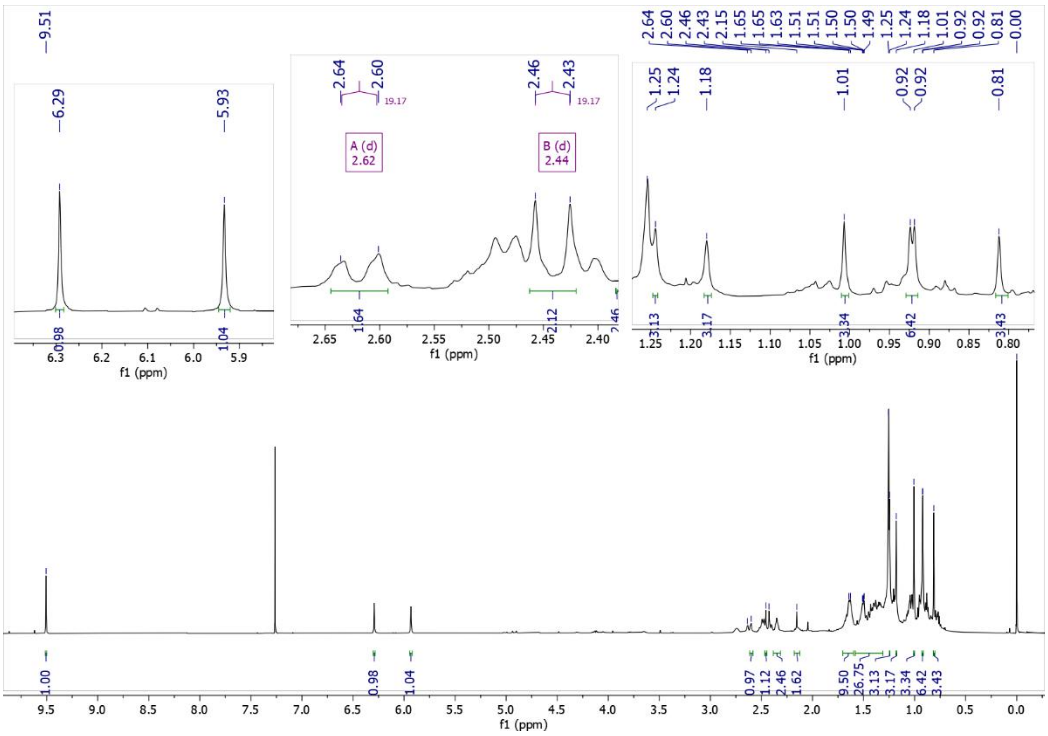

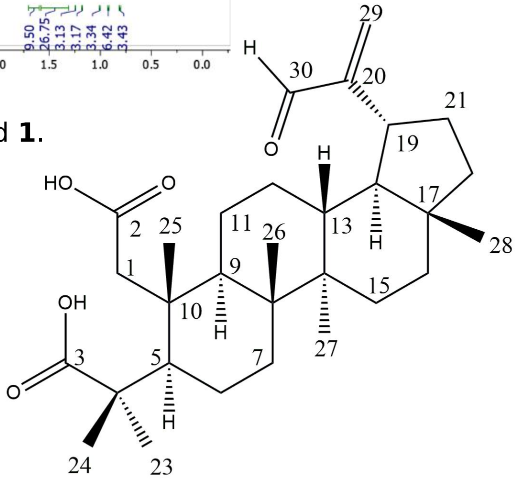

Figure S1. ^{1}H NMR spectrum (400 MHz, $CDCl_3$) of compound **1**.

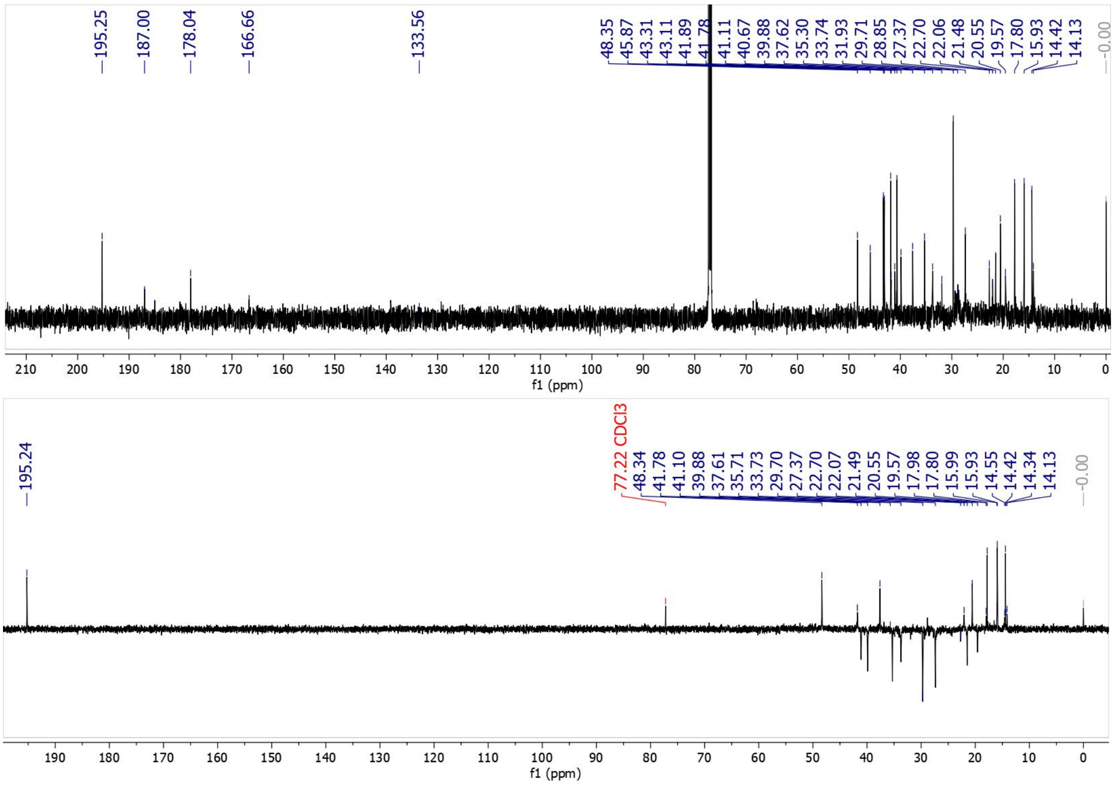

Figure S2. ^{13}C NMR and DEPT-135 spectra (100 MHz, $CDCl_3$) of compound **1**.

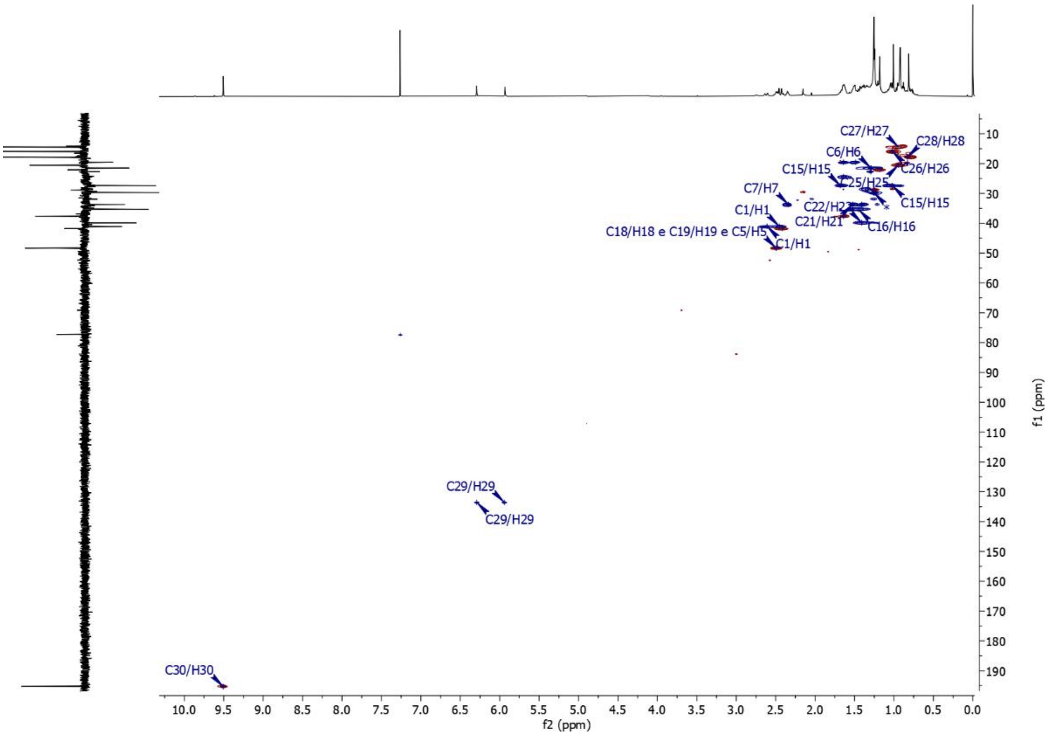

Figure S3. HSQC spectrum (600 MHz, $CDCl_3$) of compound **1**.

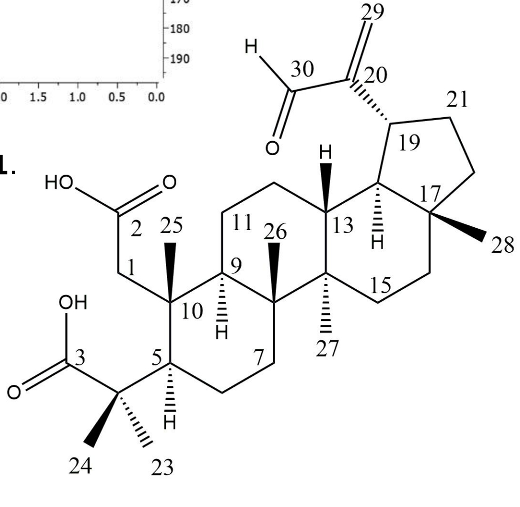

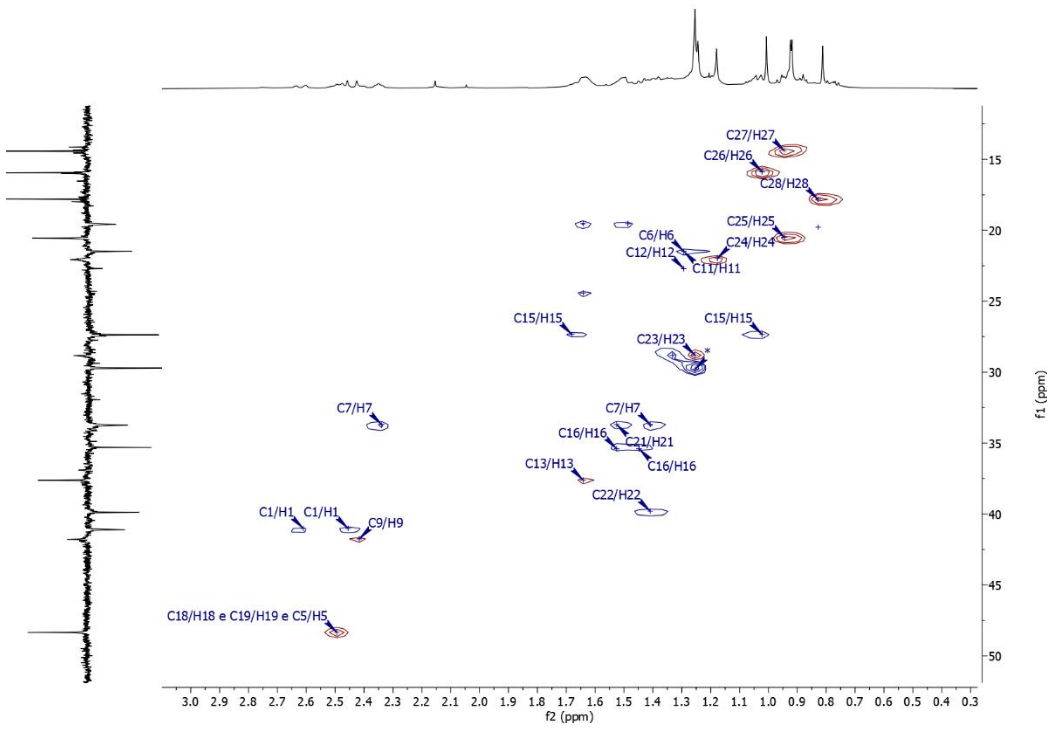

Figure S4. Expanded HSQC spectrum (600 MHz, $CDCl_3$) of compound **1** in the region between δ_H 3.0 to 0.3 ppm.

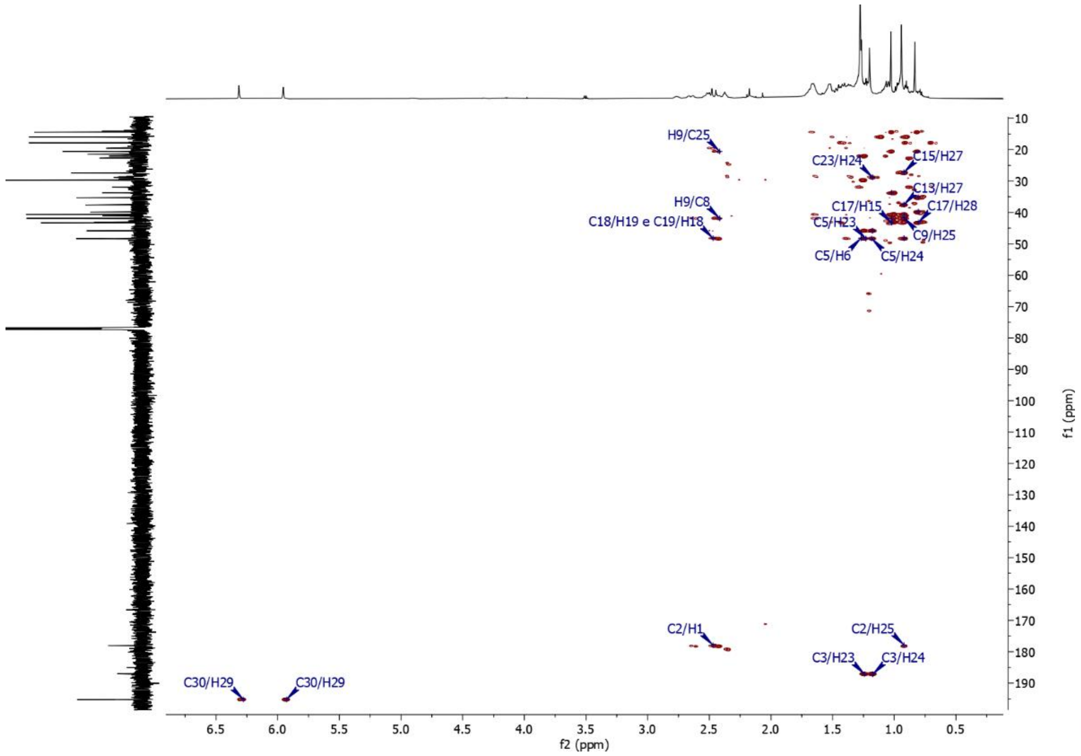

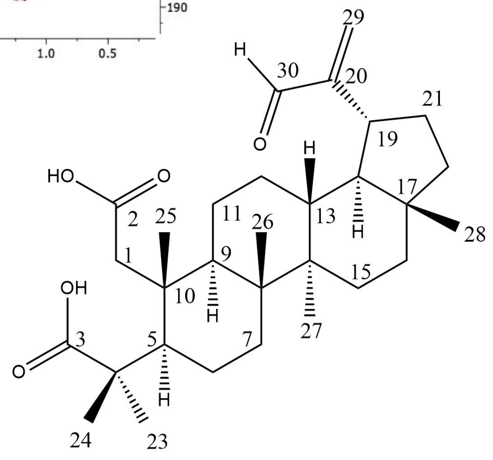

Figure S5. HMBC spectrum (600 MHz, $CDCl_3$) of compound **1**.

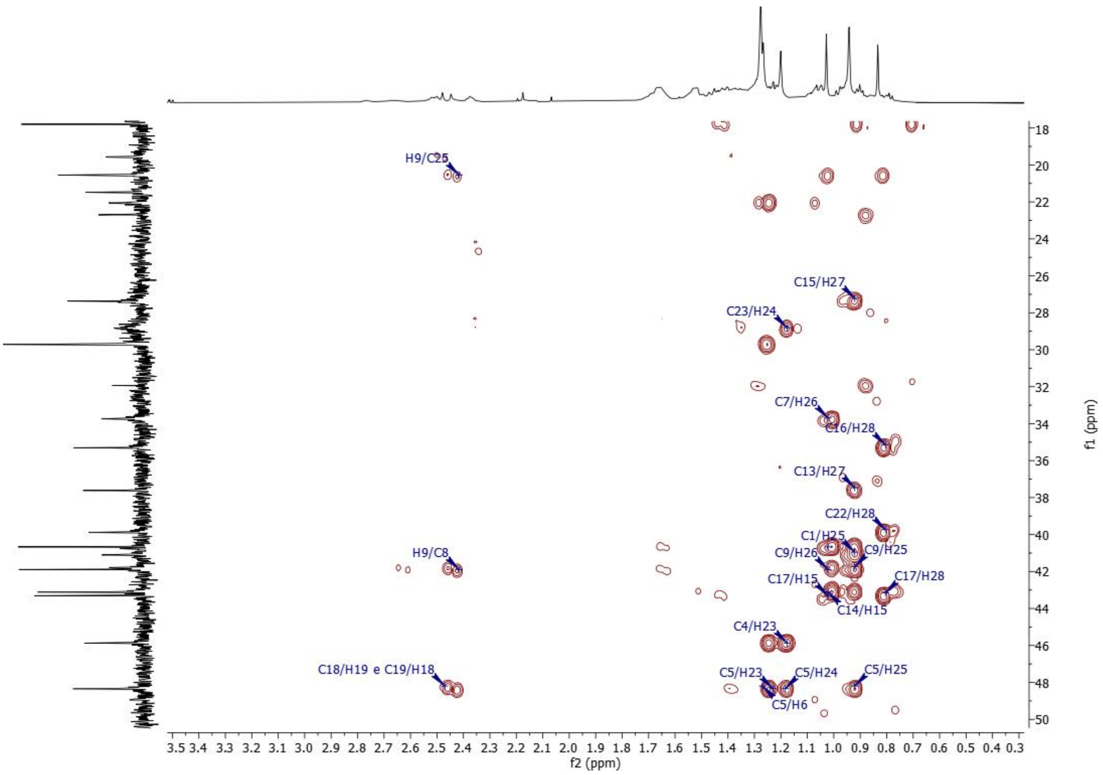

Figure S6. Expanded HMBC spectrum (600 MHz, $CDCl_3$) of compound **1** in the region between δ_H 2.7 to 0.6 ppm.

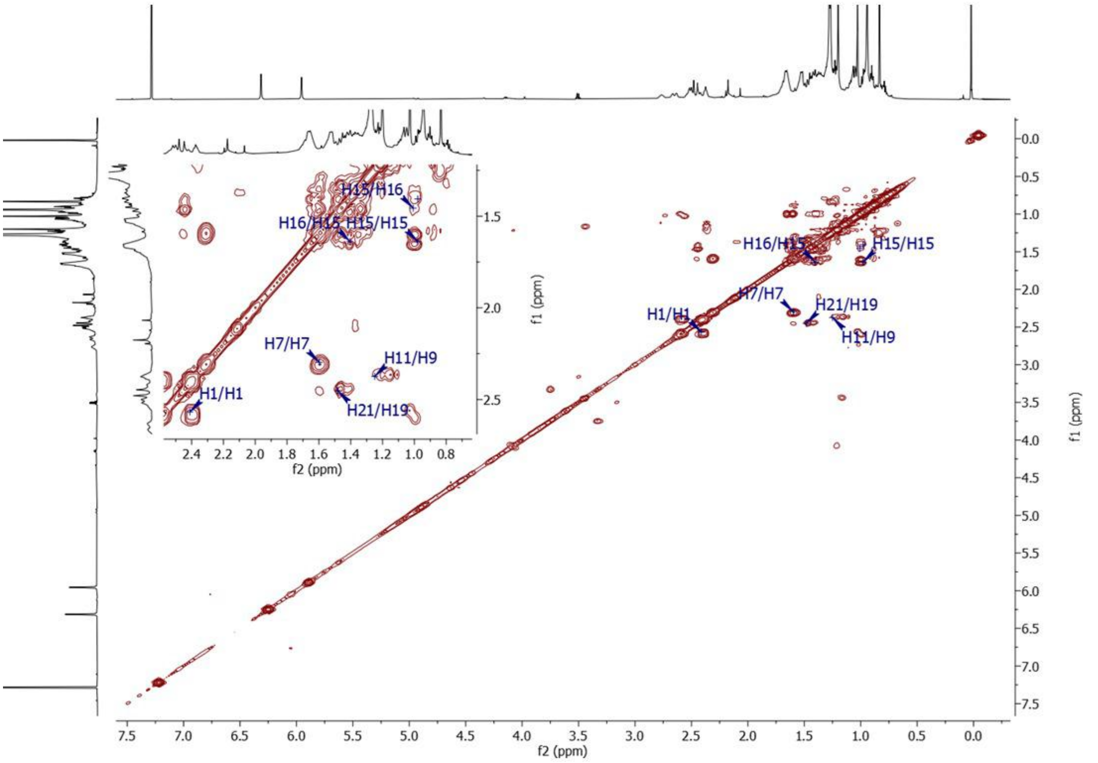

Figure S7. COSY spectrum and expanded COSY spectrum (600 MHz, $CDCl_3$) of compound **1** in the region between δ_H 2.5 to 0.8 ppm.

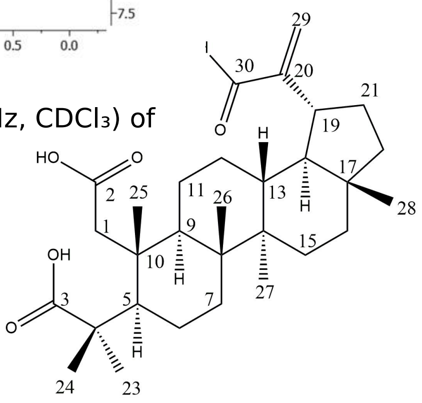

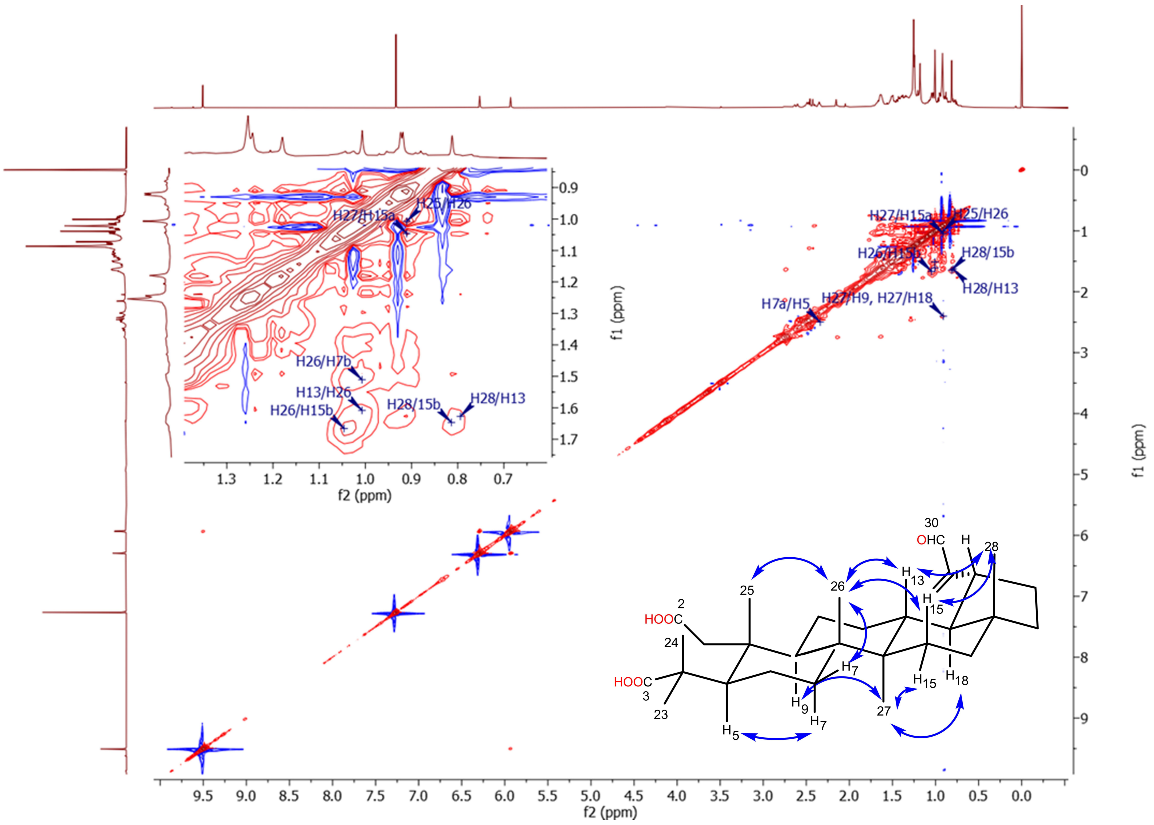

Figure S8. NOESY spectrum (600 MHz, $CDCl_3$) of compound **1.**

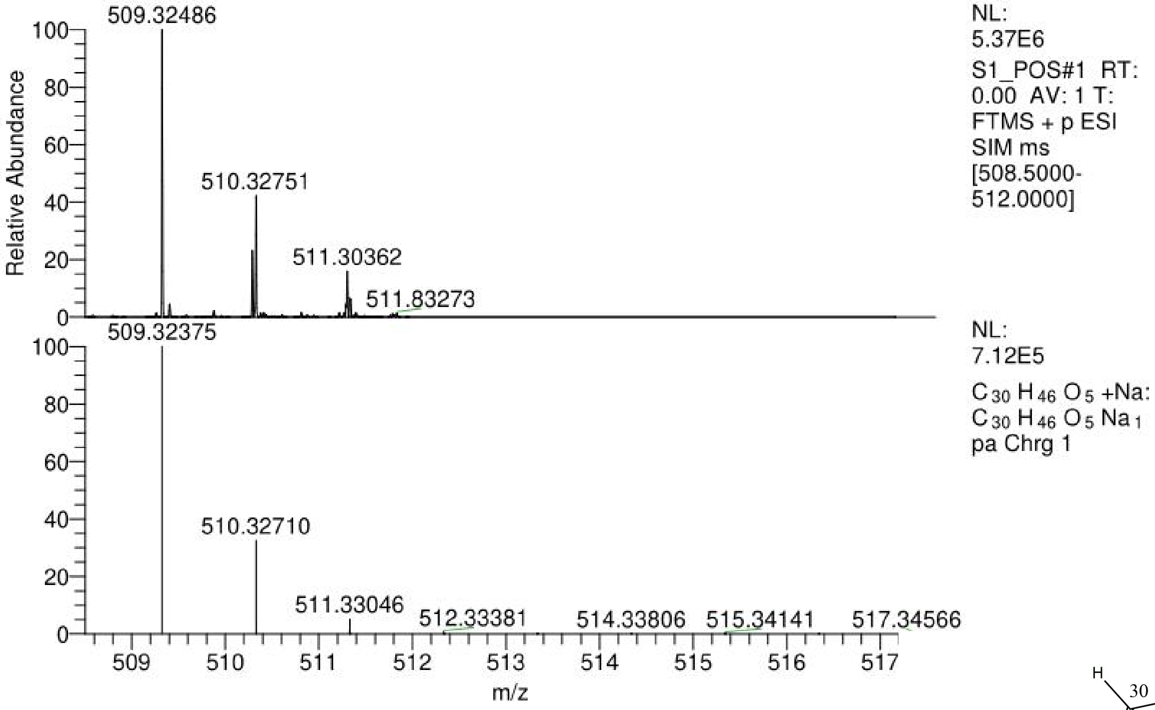

Figure S9. Mass spectrum (HR-ESI-Q-Orbitrap-MS) of compound **1**.

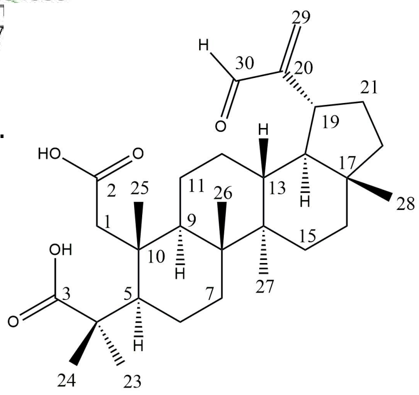

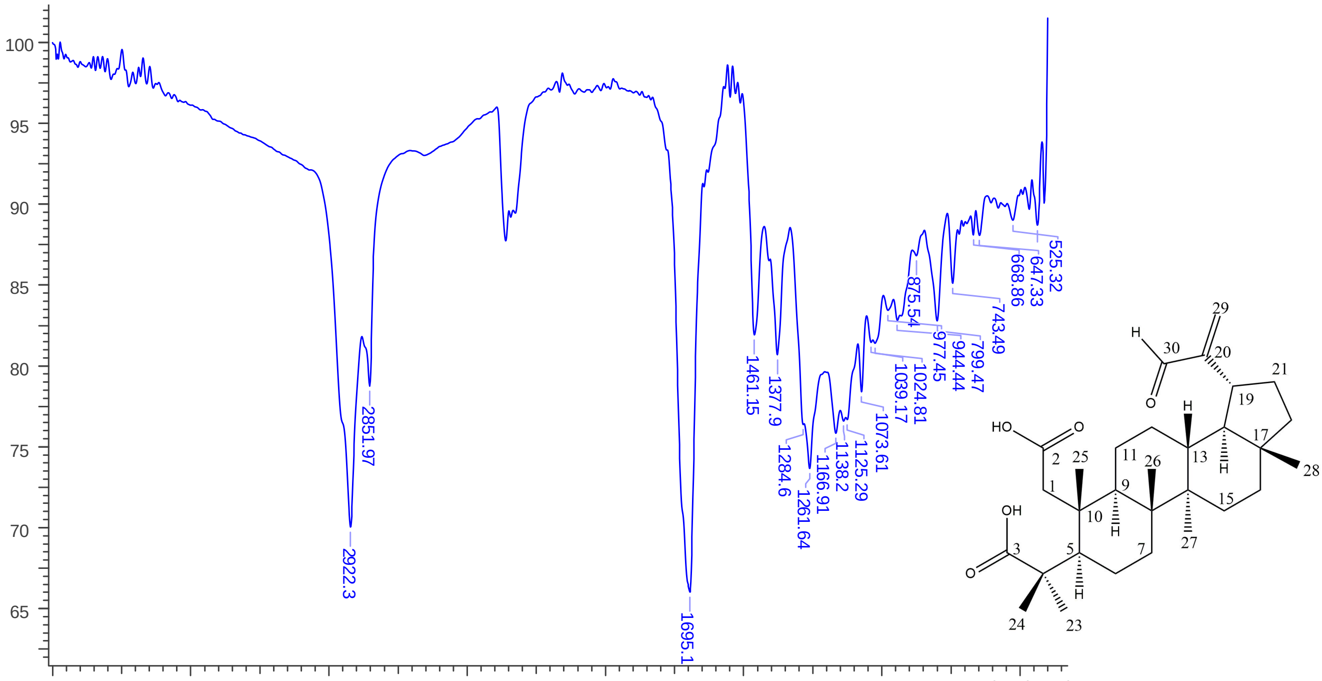

Figure S10. FTIR (ATR) spectrum of compound **1**.

In the Compound **2** spectra, unassigned correlations correspond to minor component(s) present in the mixture.

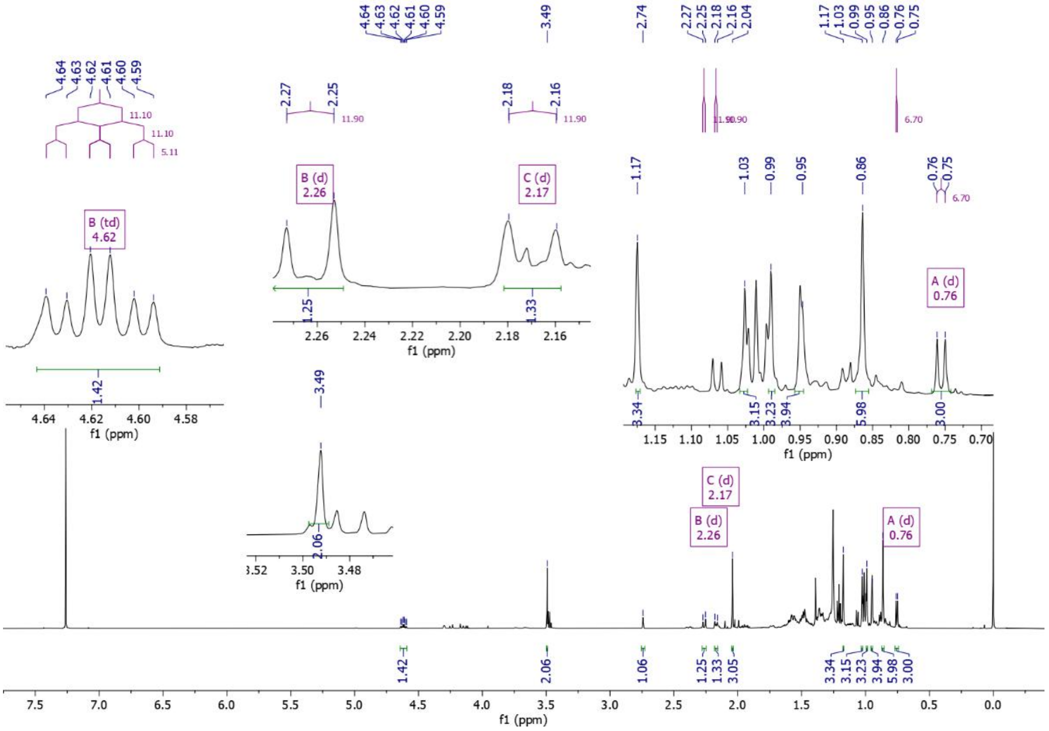

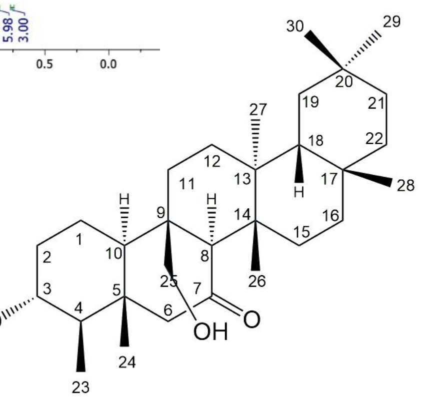

Figure S11. ^{1}H NMR spectrum (400 MHz, $CDCl_3$) of compound **2**.

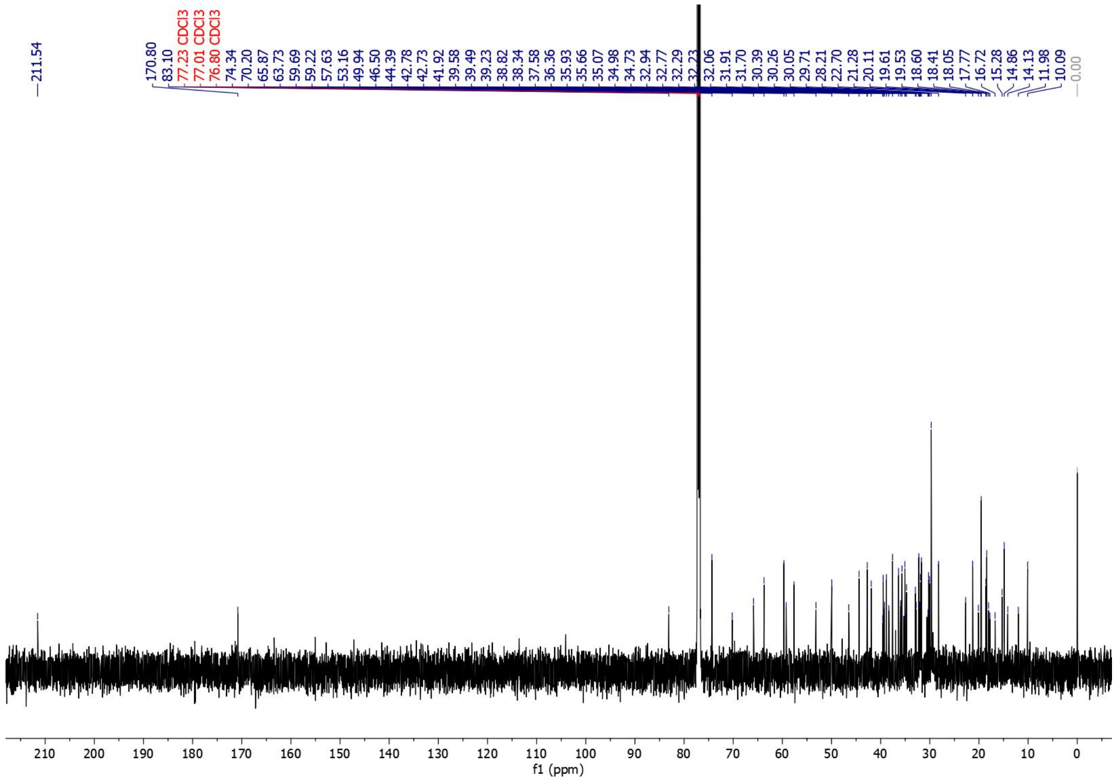

Figure S12. ^{13}C NMR and DEPT-135 spectra (100 MHz, $CDCl_3$) of compound **2**.

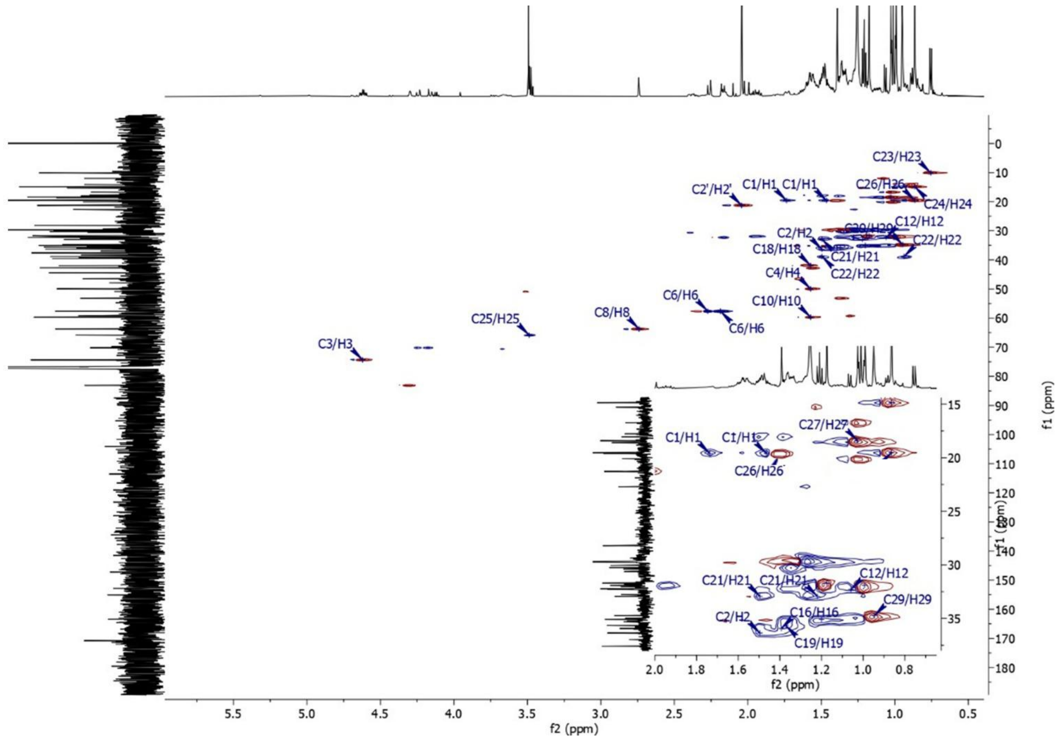

Figure S13. HSQC and expanded HSQC spectrum (600 MHz, $CDCl_3$) of compound **2** in the region between δ_H 2.0 to 0.8 ppm.

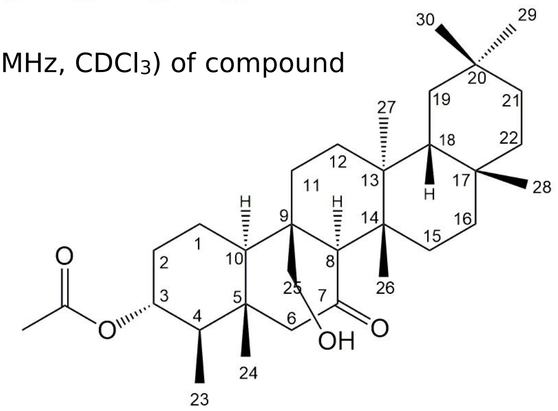

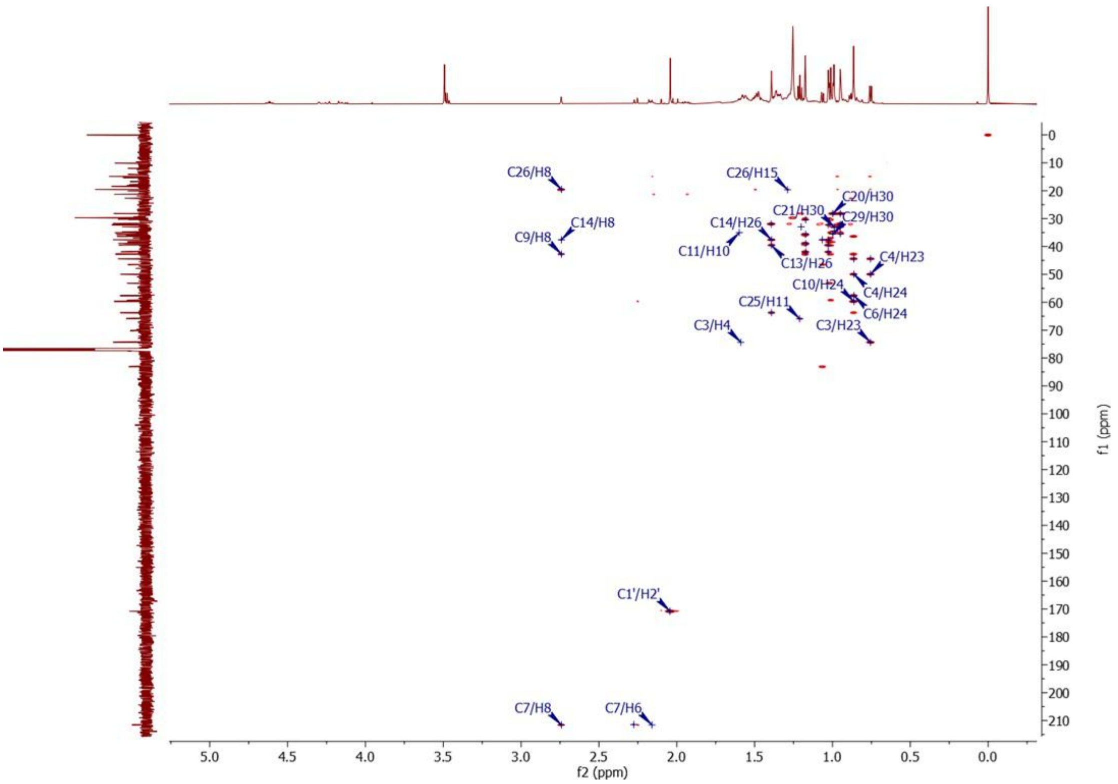

Figure S14. HMBC spectrum (600 MHz, $CDCl_3$) of compound **2**.

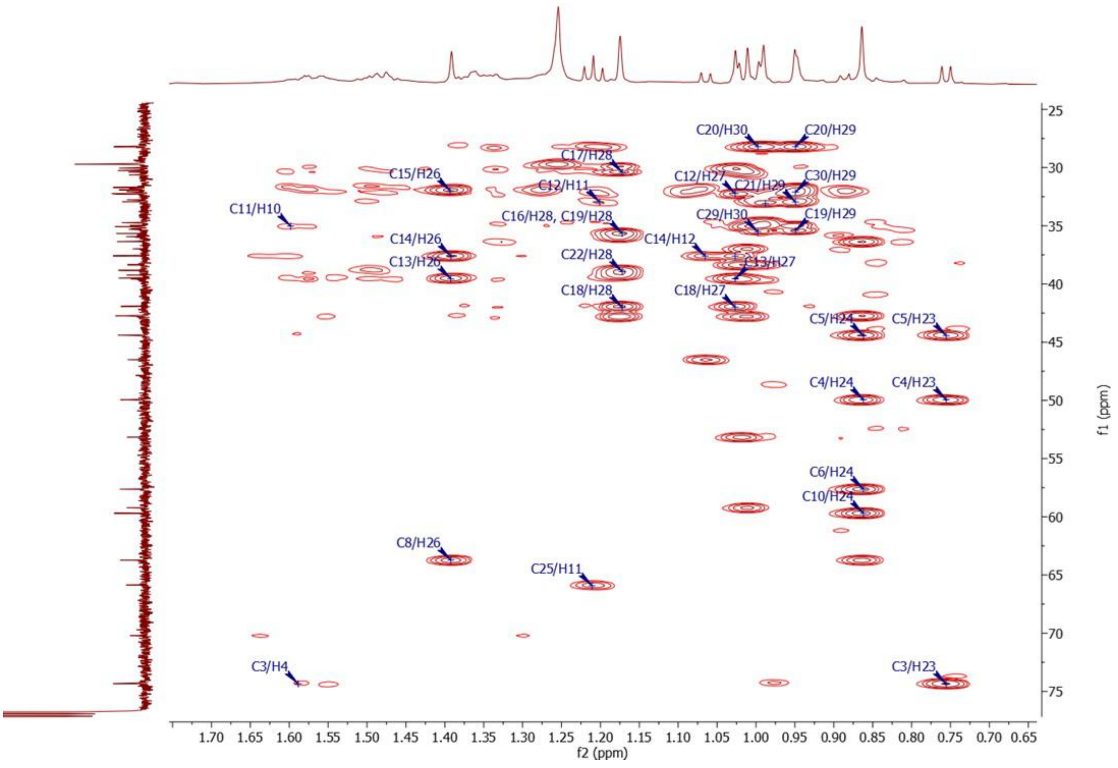

Figure S15. Expanded HMBC spectrum (600 MHz, $CDCl_3$) of compound **2** in the region between δ_H 2.0 to 0.6 ppm.

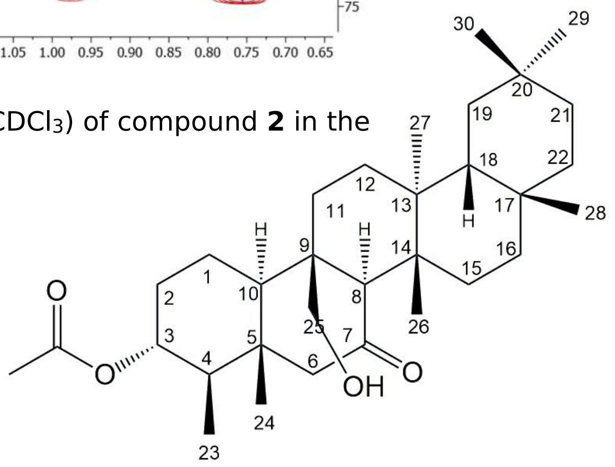

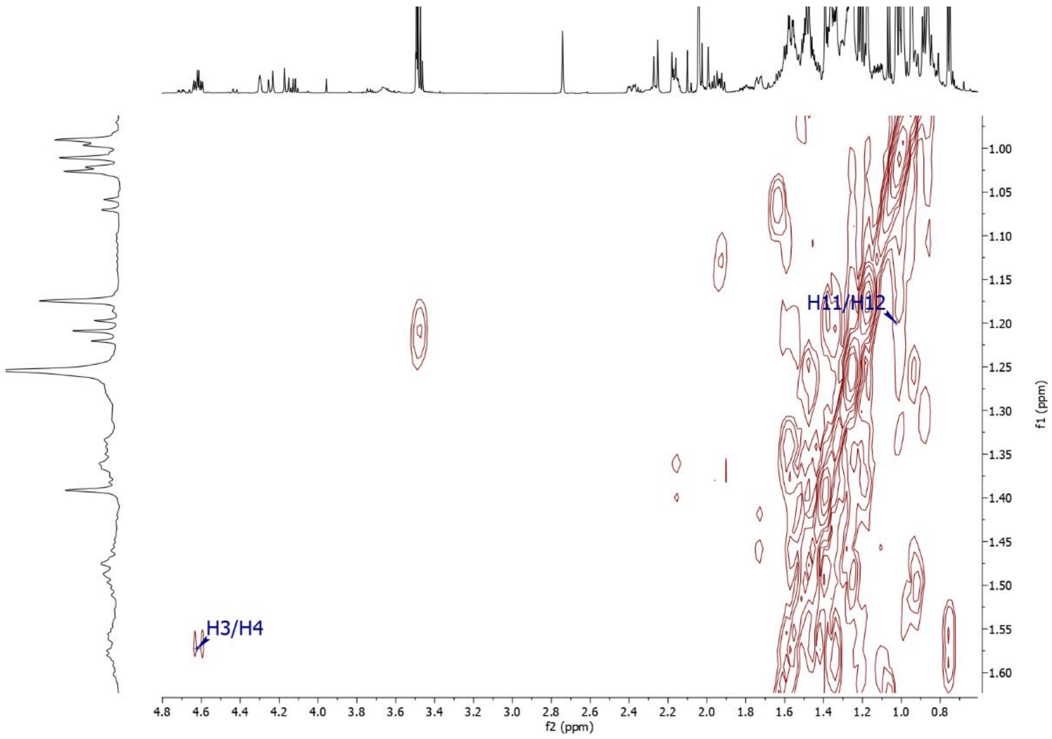

Figure S16. COSY spectrum (600 MHz, $CDCl_3$) of compound **2**.

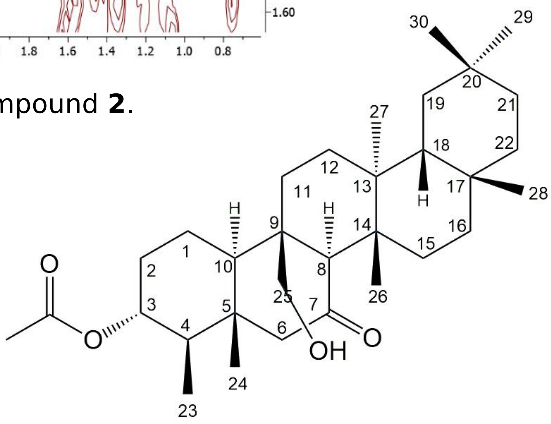

Table S1. ^{13}C NMR (100 MHz, $CDCl_3$) data of compounds **3**, **4** and **5** and comparison with the literature data of friedelan-3-one, friedelan-3*β*-ol and friedelan-3*α*-ol.

**Atom**	***δ*C 3**	***δ*C friedelan-3-one**[1]	***δ*C 4**	***δ*C friedelan-3*β*-ol**[2]	***δ*C 5**	***δ*C friedelan-3*α*-ol**[2]
**1**	22.4	22.3	15.9	16.16	19.7	19.76
**2**	41.7	41.5	36.2	36.14	37.2	37.00
**3**	213.4	213.2	72.9	71.59	72.4	71.11
**4**	58.4	58.2	49.3	49.62	53.3	53.41
**5**	42.3	42.1	38.0	38.09	38.3	38.12
**6**	41.4	41.3	41.9	41.99	41.5	41.53
**7**	18.4	18.2	17.7	17.69	18.0	17.92
**8**	53.3	53.1	53.3	53.27	53.1	53.00
**9**	37.6	37.4	37.2	37.18	36.8	37.05
**10**	59.6	59.4	61.5	61.65	60.2	60.25
**11**	35.8	35.6	35.5	35.66	35.7	35.57
**12**	30.7	30.5	30.8	30.69	30.7	30.64
**13**	39.9	39.7	38.5	38.38	38.4	38.31
**14**	38.5	38.3	39.8	39.69	39.8	39.70
**15**	32.6	32.4	32.5	32.34	33.0	32.38
**16**	36.2	36.0	35.7	35.90	36.2	36.12
**17**	30.1	30.0	30.2	30.02	30.2	30.01
**18**	42.9	42.8	43.0	42.88	43.0	42.88
**19**	35.5	35.3	35.3	35.36	35.5	35.36
**20**	28.3	28.1	28.3	28.17	28.3	28.16
**21**	32.9	32.7	33.0	32.88	32.5	32.88
**22**	39.4	39.2	39.4	39.28	39.4	39.27
**23**	7.0	6.8	11.8	12.09	10.1	10.22
**24**	14.8	14.6	16.5	16.58	14.7	14.63
**25**	18.1	17.9	18.4	18.35	18.3	18.17
**26**	20.4	20.2	20.3	20.13	20.3	20.18
**27**	18.8	18.6	18.8	18.69	18.8	18.68
**28**	32.2	32.1	32.2	32.07	32.2	32.13
**29**	35.2	35.0	35.2	35.02	35.2	35.02
**30**	31.9	31.8	31.9	31.78	31.9	31.86

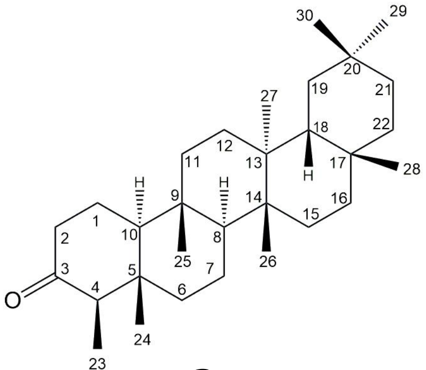

3

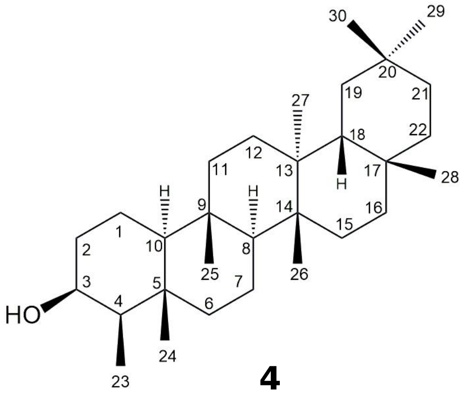

4

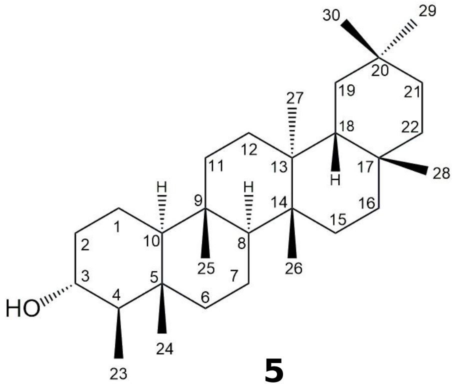

5

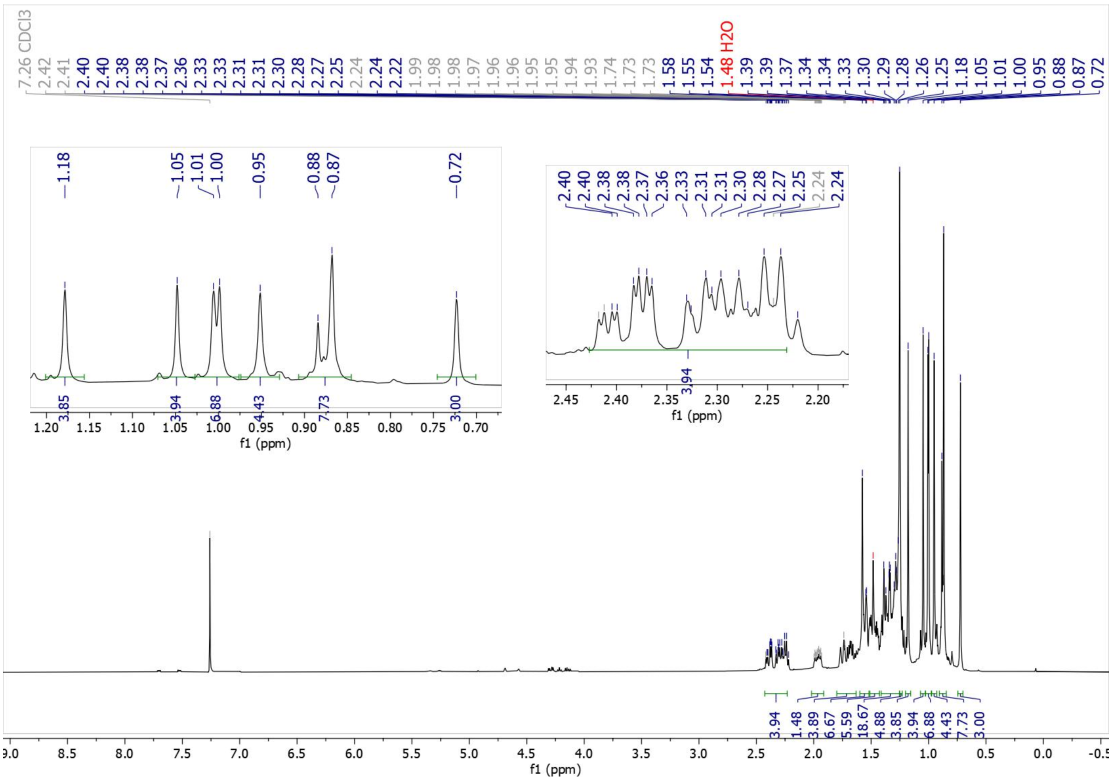

Figure S17. ^{1}H NMR spectrum (400 MHz, $CDCl_3$) of compound **3**.

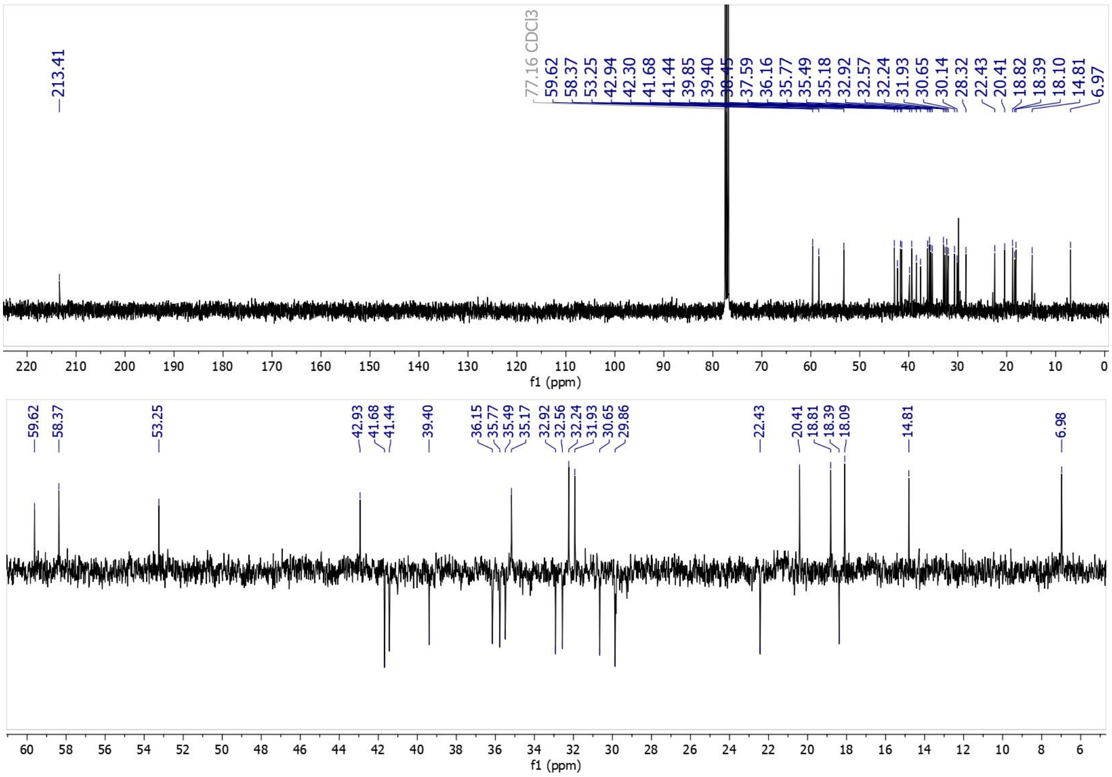

Figure S18. ^{13}C NMR and DEPT-135 spectra (100 MHz, $CDCl_3$) of compound **3**.

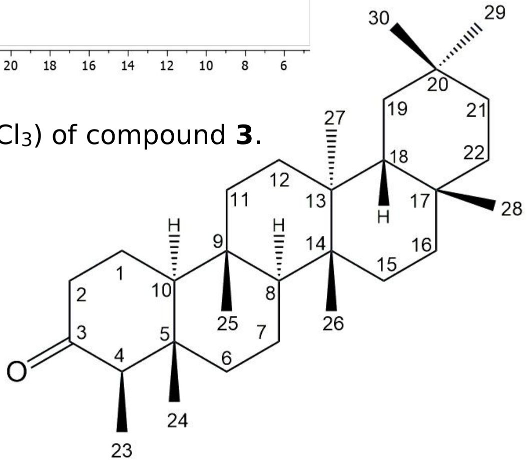

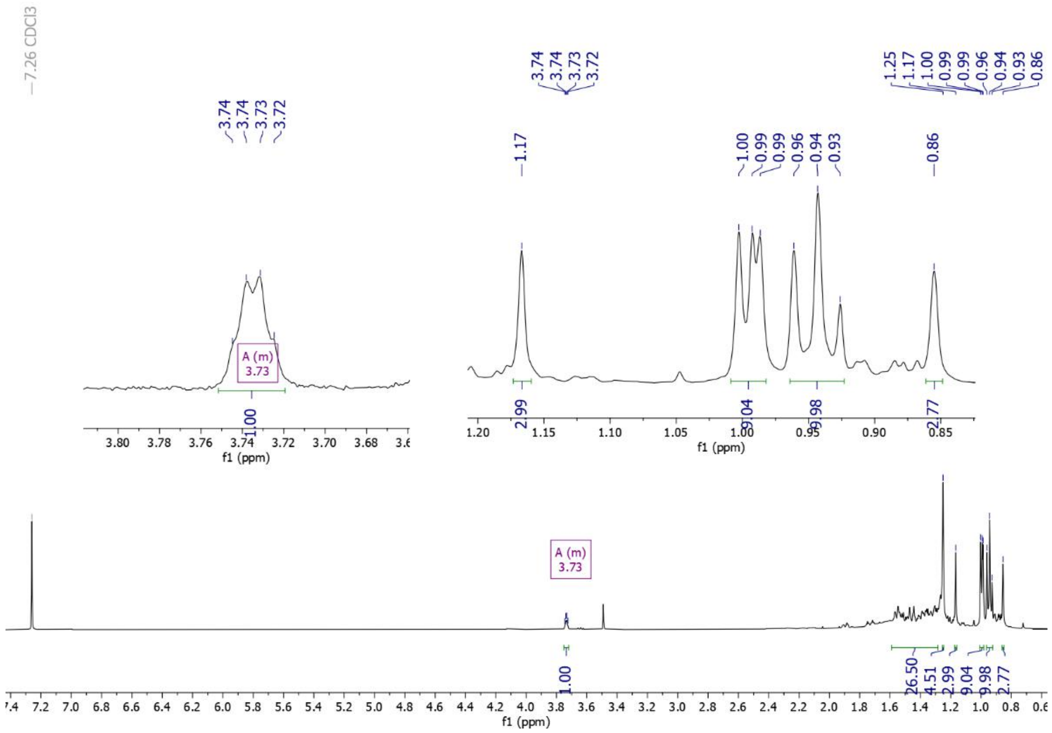

Figure S19. ^{1}H NMR spectrum (400 MHz, $CDCl_3$) of compound **4**.

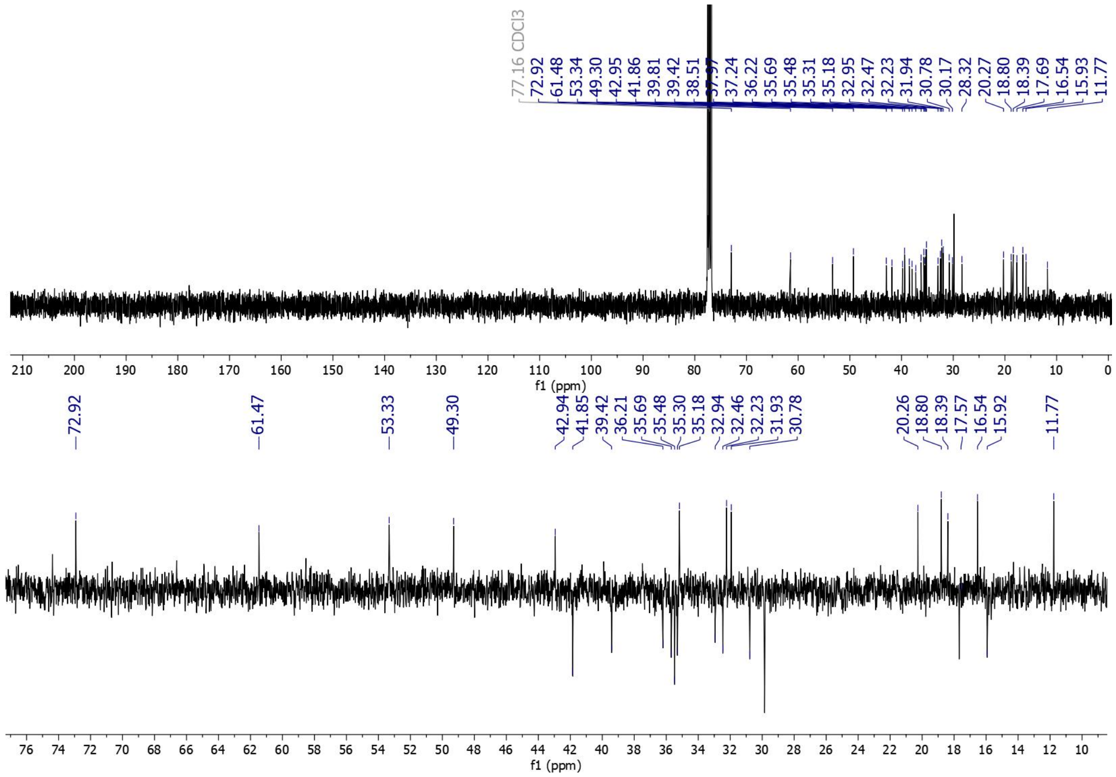

Figure S20. ^{13}C NMR and DEPT-135 spectra (100 MHz, $CDCl_3$) of compound **4**.

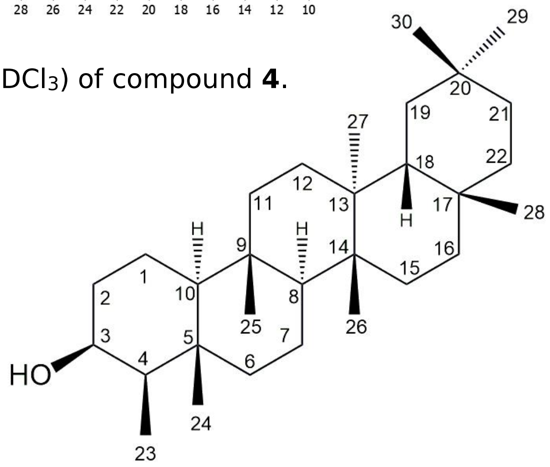

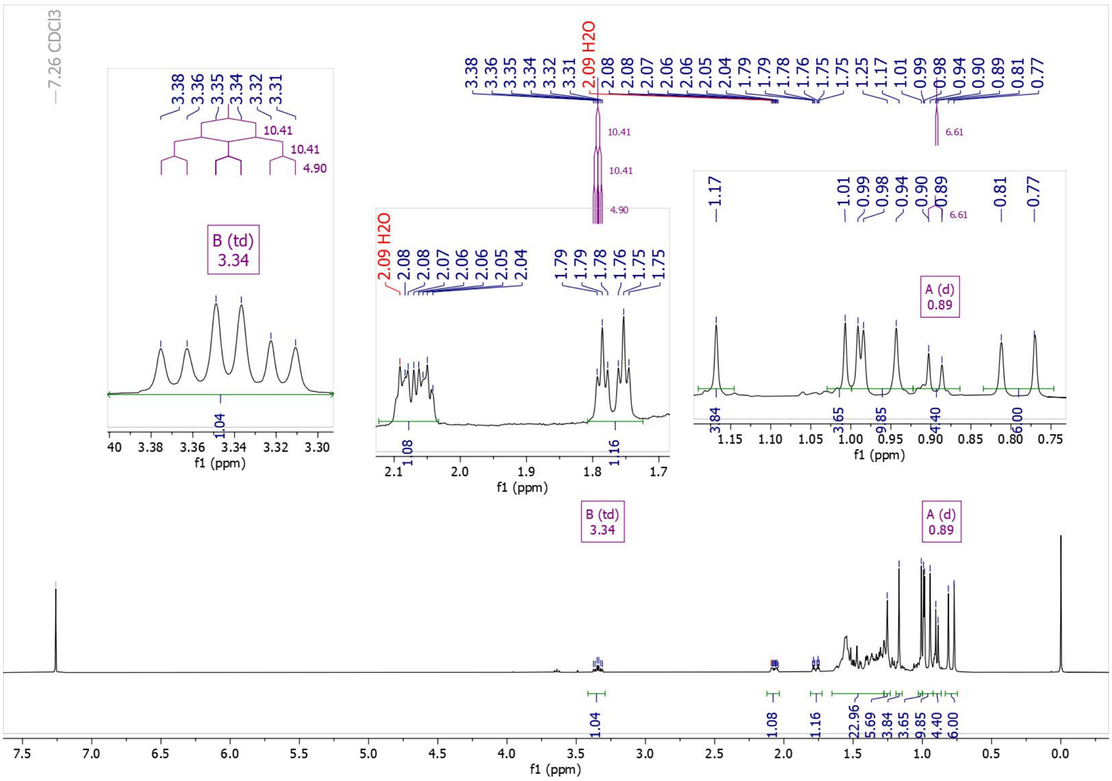

Figure S21. ^{1}H NMR spectrum (400 MHz, $CDCl_3$) of compound **5**.

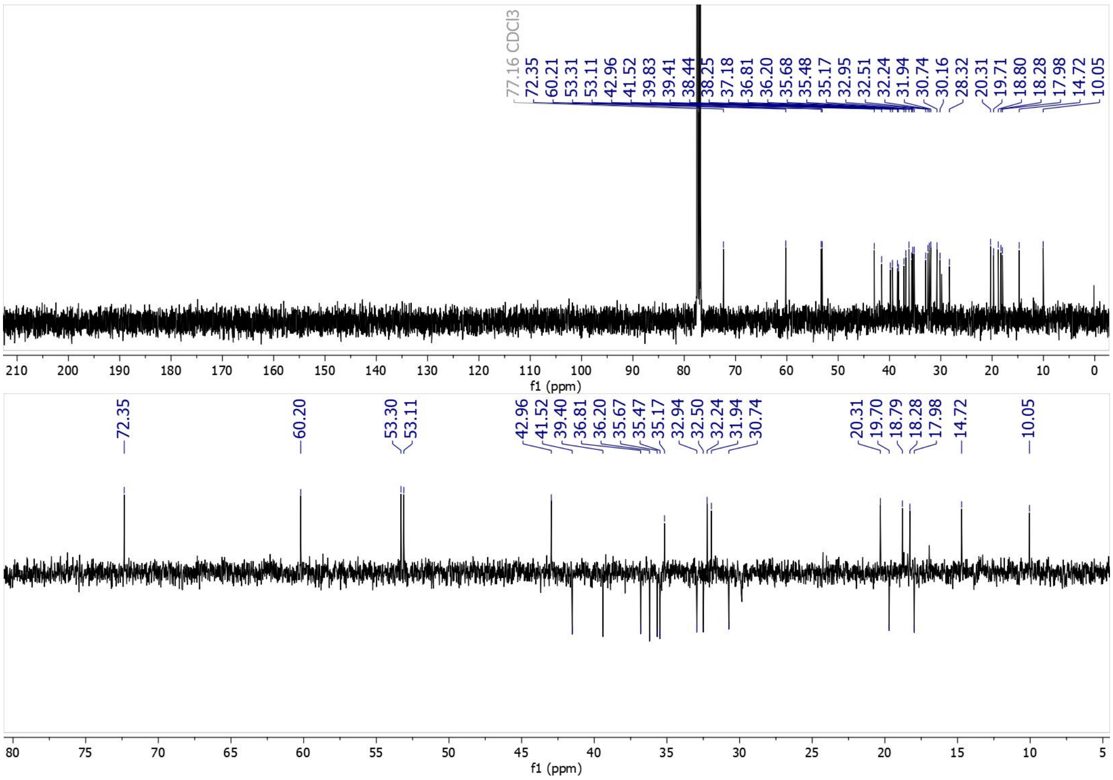

Figure S22. ^{13}C NMR and DEPT-135 spectra (100 MHz, $CDCl_3$) of compound **5**.

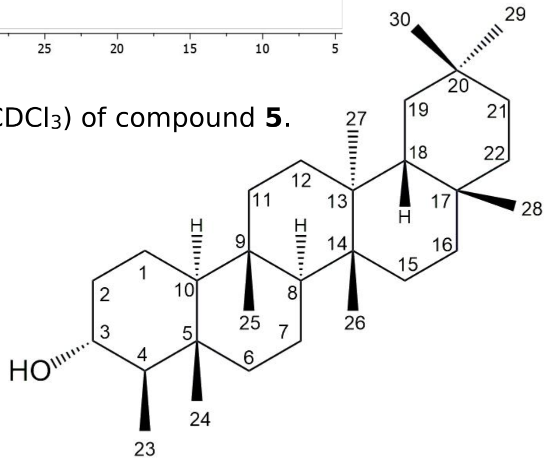

Table S2. ^{13}C NMR (100 MHz, $CDCl_3$) data of compounds **6** and **7** and comparison with the literature data of *β*-sitosteryl palmitate and lupenyl stearate.

Atom	δ_C 6	δ_C *β*-sitosteryl palmitate[3]	Atom	δ_C 7	δ_C lupenyl stearate[4]
**1**	37.2	37.0	**1**	38.0	37.8
**2**	32.1	31.9	**2**	23.9	23.4
**3**	73.8	73.7	**3**	80.8	80.7
**4**	38.3	38.2	**4**	38.5	38.5
**5**	139.9	139.7	**5**	55.5	55.5
**6**	122.7	122.6	**6**	18.4	18.3
**7**	39.9	39.7	**7**	34.4	34.3
**8**	32.0	31.9	**8**	41.0	40.9
**9**	50.2	50.0	**9**	50.5	50.4
**10**	36.8	36.2	**10**	38.2	38.1
**11**	21.2	21.0	**11**	21.1	21.0
**12**	34.9	34.7	**12**	25.3	25.2
**13**	42.5	42.3	**13**	37.2	37.2
**14**	56.8	56.7	**14**	43.0	42.7
**15**	24.5	24.3	**15**	27.6	27.5
**16**	28.4	28.2	**16**	35.7	35.6
**17**	56.2	56.0	**17**	43.2	43.0
**18**	12.1	12.0	**18**	48.4	48.4
**19**	19.2	19.3	**19**	48.2	48.0
**20**	36.3	36.6	**20**	151.1	150.8
**21**	18.9	18.8	**21**	29.5	29.4
**22**	34.1	34.0	**22**	40.2	40.0
**23**	26.2	26.1	**23**	28.0	27.5
**24**	46.0	45.9	**24**	16.7	16.3
**25**	29.5	29.5	**25**	16.3	16.3
**26**	20.0	19.8	**26**	16.1	16.3
**27**	19.5	19.0	**27**	14.7	14.5
**28**	23.2	23.1	**28**	18.2	18.3
**29**	12.0	11.9	**29**	109.5	109.4
**1'**	173.5	173.3	**30**	19.4	19.4
**2' – 11'**	22.9 - 29.9	22.7 - 29.7	**1'**	173.9	173.5
**12'**	14.3	14.1	**2'**	35.0	34.8
**-**			**3'**	32.1	31.9
**-**			**4'**	22.9	22.7
**-**			**5'**	25.3	25.2
**-**			**6' - 17'**	29.3 - 29.9	29.2 - 29.7
**-**			**18'**	14.3	14.1

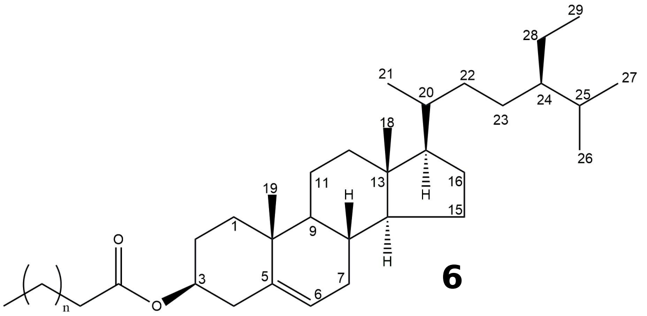

6

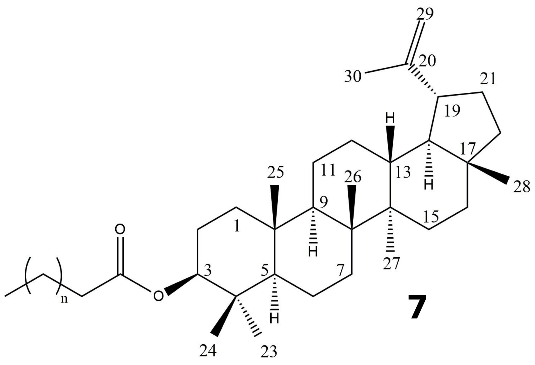

7

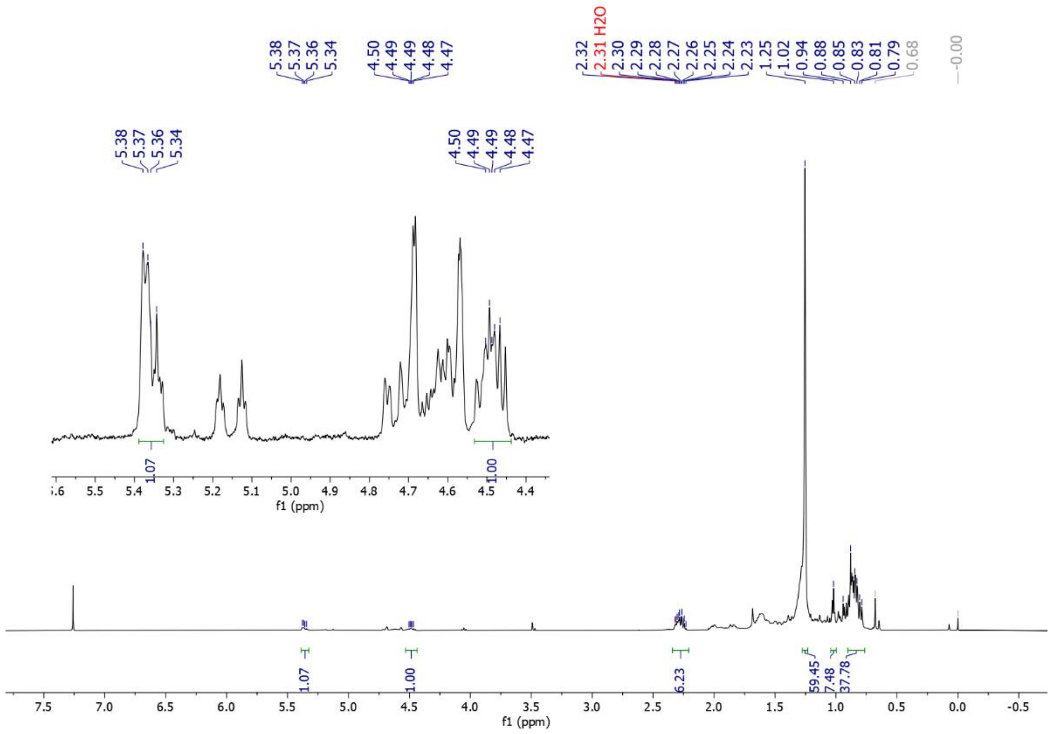

Figure S23. ^{1}H NMR spectrum (400 MHz, $CDCl_3$) of compound **6**.

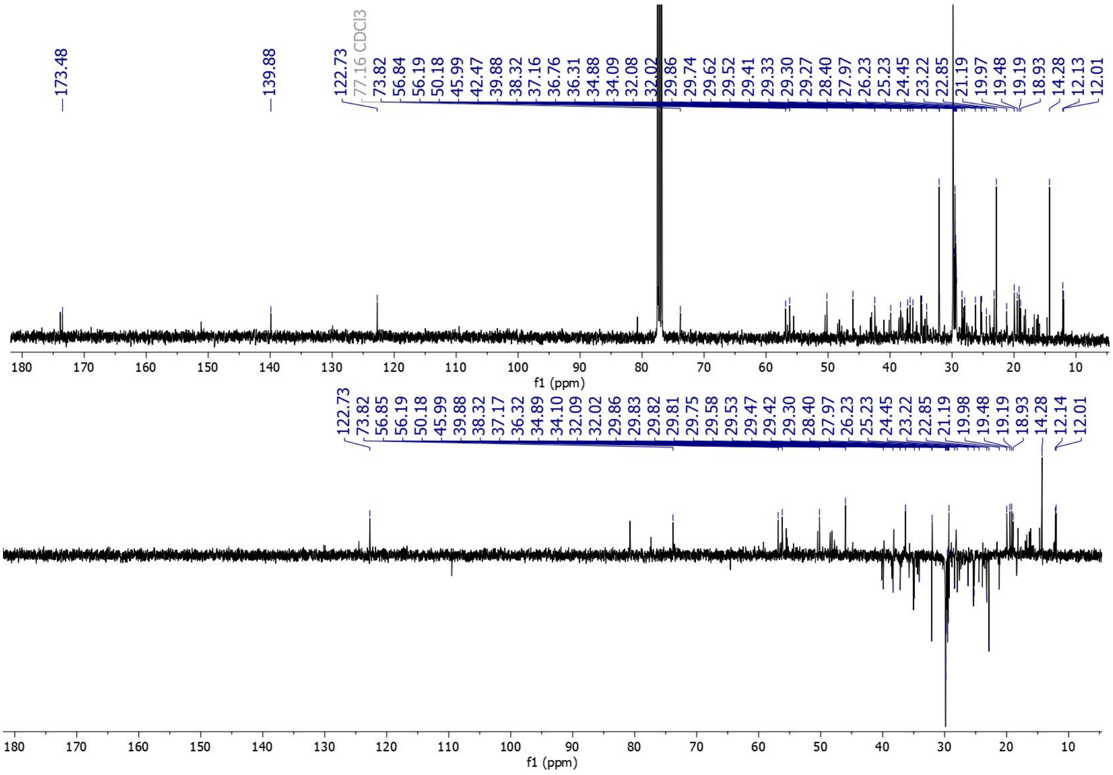

Figure S24. ^{13}C NMR and DEPT-135 spectra (100 MHz, $CDCl_3$) of compound **6**.

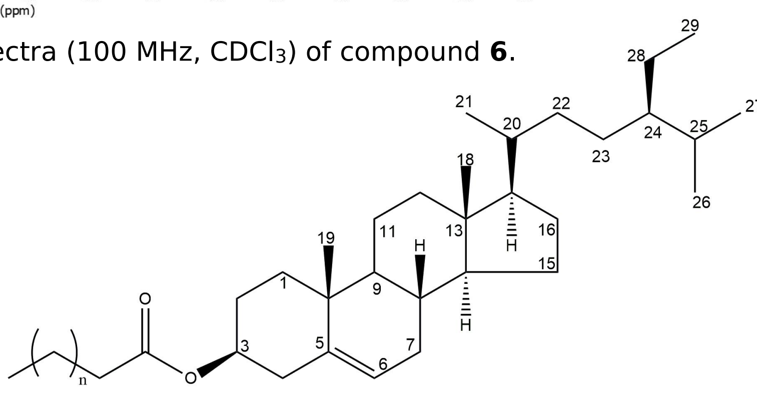

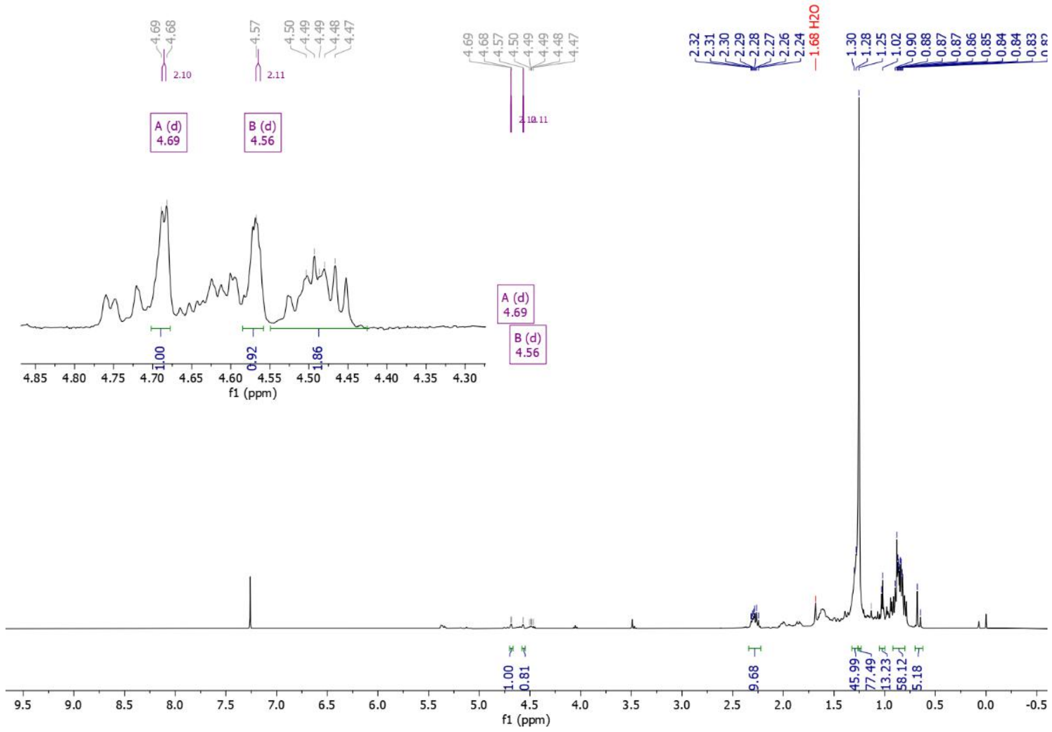

Figure S25. ^{1}H NMR spectrum (400 MHz, $CDCl_3$) of compound **7**.

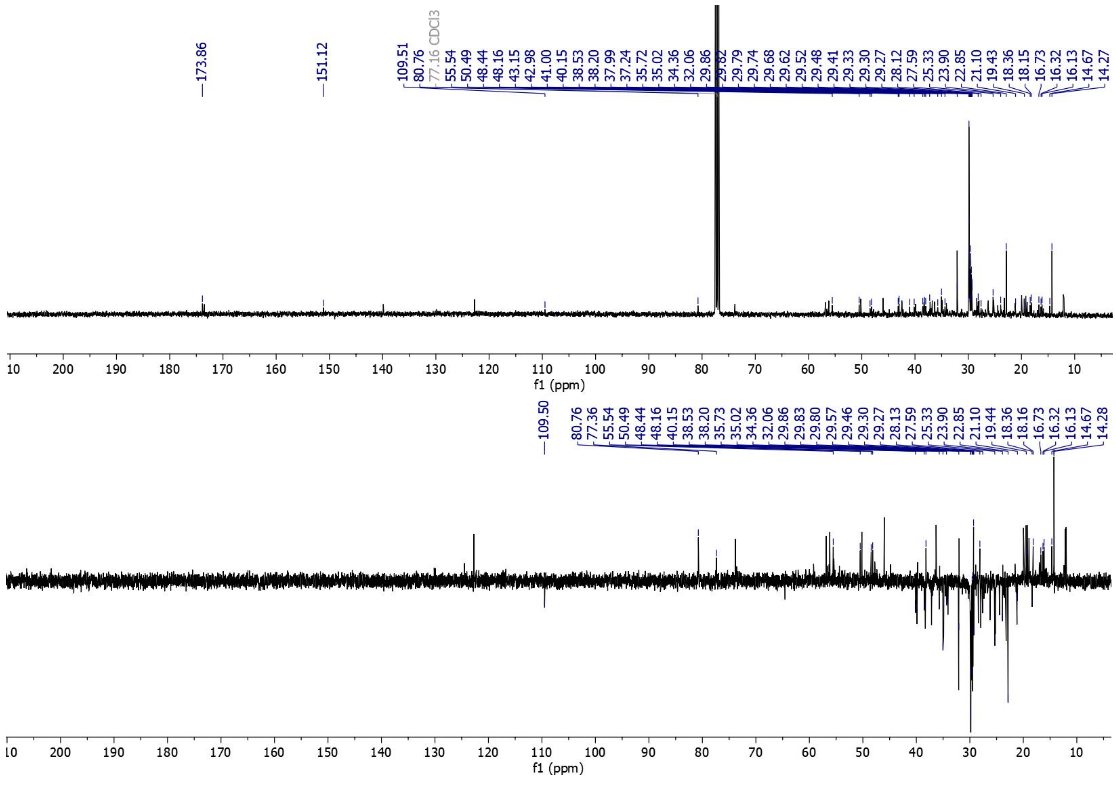

Figure S26. ^{13}C NMR and DEPT-135 spectra (100 MHz, $CDCl_3$) of compound **7**.

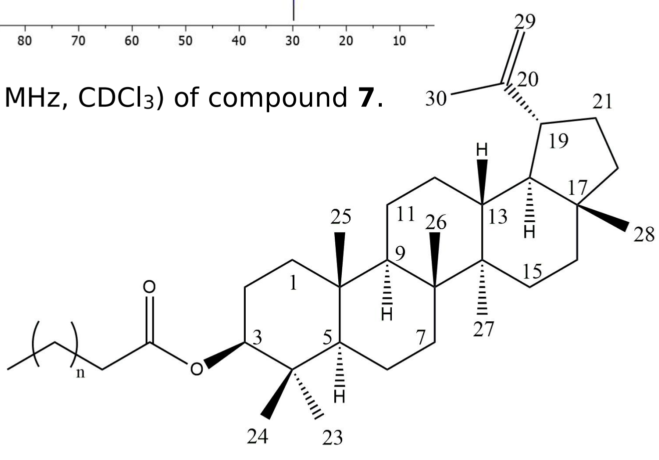

Table S3. ^{13}C NMR (100 MHz, $CDCl_3$) data of compounds **8** and **9** and comparison with the literature data of friedelan-3,7-dione and 2,3-seco-lup-20(29)-ene-2,3-dioic acid.

**Atom**	***δ*C 8**	***δ*C friedelan-3,7-dione**[5]	***δ*C 9**	***δ*C 2,3-seco-lup-20(29)-ene-2,3-dioic acid**[6]
**1**	21.8	21.6	40.9	40.9
**2**	41.0	40.8	178.1	178.5
**3**	211.2	210.6	187.1	187.5
**4**	58.0	57.8	46.0	45.6
**5**	47.7	47.0	48.4	48.2
**6**	57.0	56.9	21.7	21.3
**7**	210.6	210.2	33.7	33.7
**8**	63.6	63.4	42.0	41.8
**9**	42.5	42.4	41.3	41.7
**10**	59.2	59.0	40.2	40.7
**11**	35.6	35.5	19.4	19.2
**12**	29.7	29.8	25.2	24.9
**13**	39.5	39.4	38.1	37.9
**14**	37.6	37.5	43.4	43.2
**15**	31.7	31.6	27.6	27.5
**16**	36.4	36.3	35.6	35.5
**17**	30.0	30.1	43.2	43.2
**18**	41.9	41.8	48.6	48.4
**19**	35.1	34.9	48.2	48.0
**20**	28.2	28.0	151.1	150.9
**21**	32.9	32.8	29.9	29.8
**22**	38.8	38.6	40.1	39.9
**23**	7.0	6.8	29.9	29.8
**24**	15.3	15.1	21.7	21.3
**25**	18.4	18.2	20.7	20.8
**26**	19.4	19.2	16.1	15.9
**27**	19.6	19.4	14.7	14.6
**28**	32.3	32.1	18.1	18.0
**29**	31.9	31.8	109.6	109.4
**30**	34.7	34.6	40.9	40.9

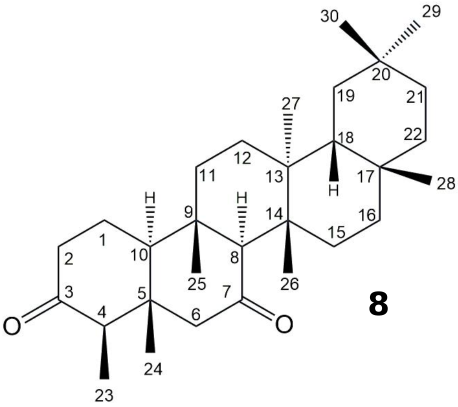

8

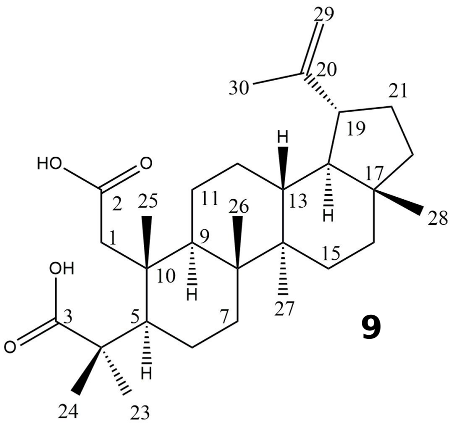

9

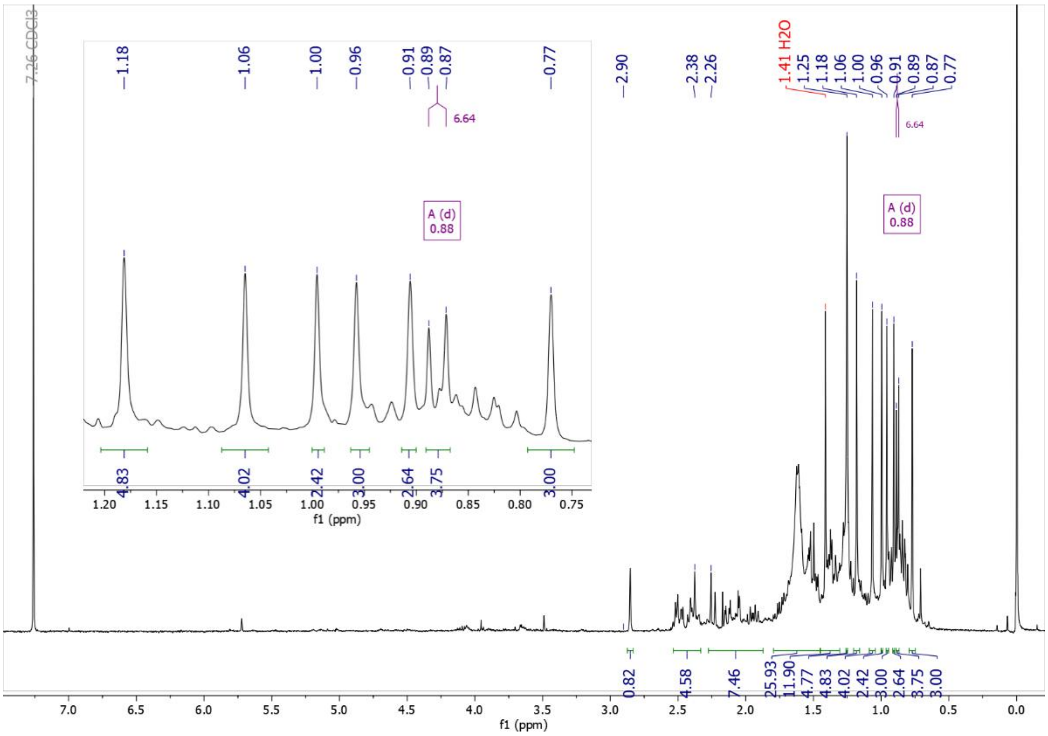

Figure S27. ^{1}H NMR spectrum (400 MHz, $CDCl_3$) of compound **8**.

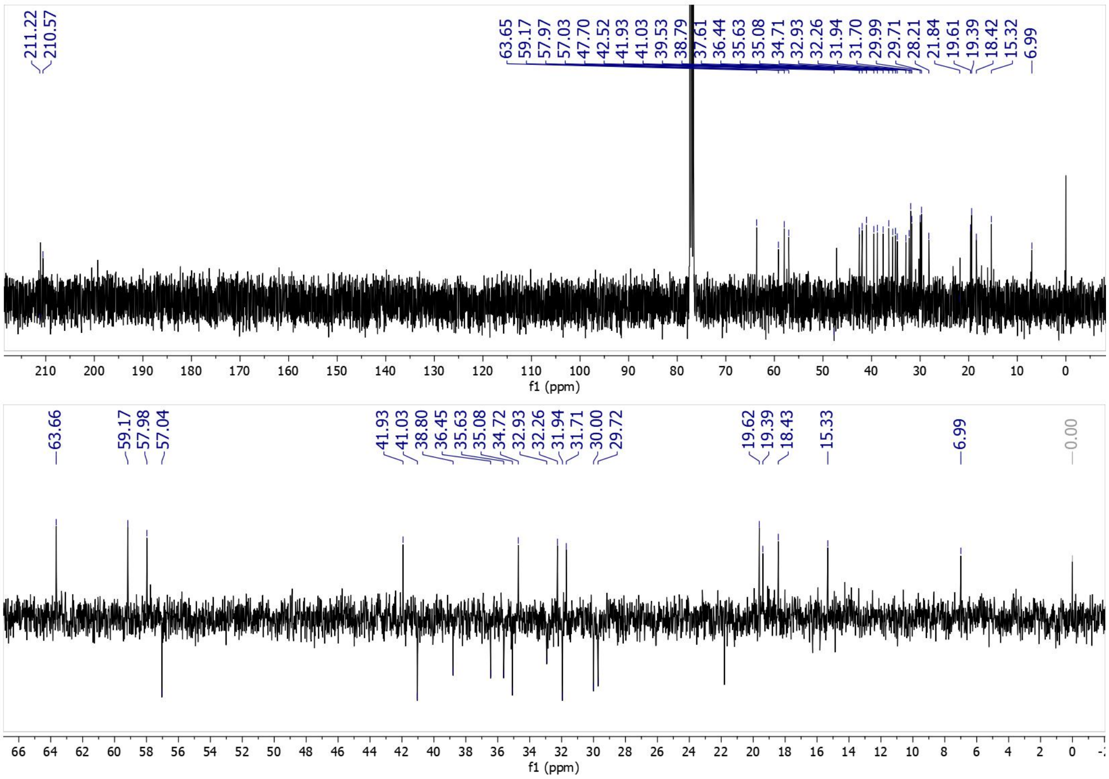

Figure S28. ^{13}C NMR and DEPT-135 spectra (100 MHz, $CDCl_3$) of compound **8**.

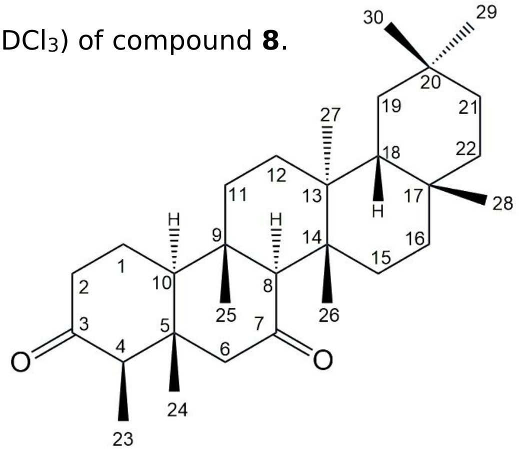

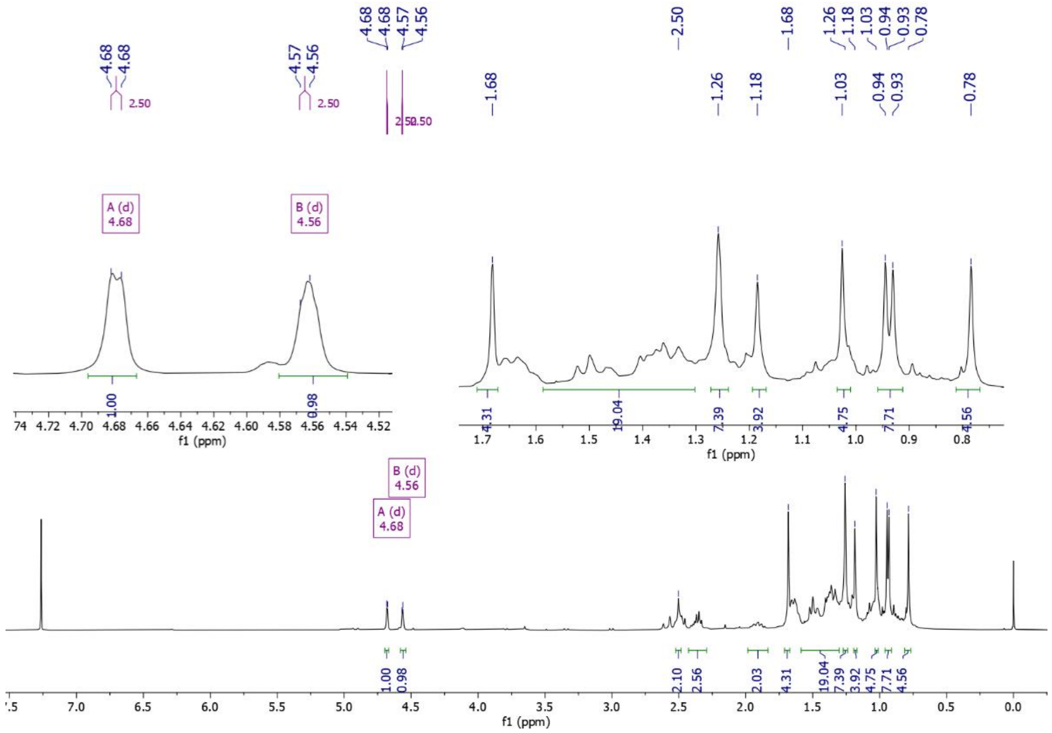

Figure S29. ^{1}H NMR spectrum (400 MHz, $CDCl_3$) of compound **9**.

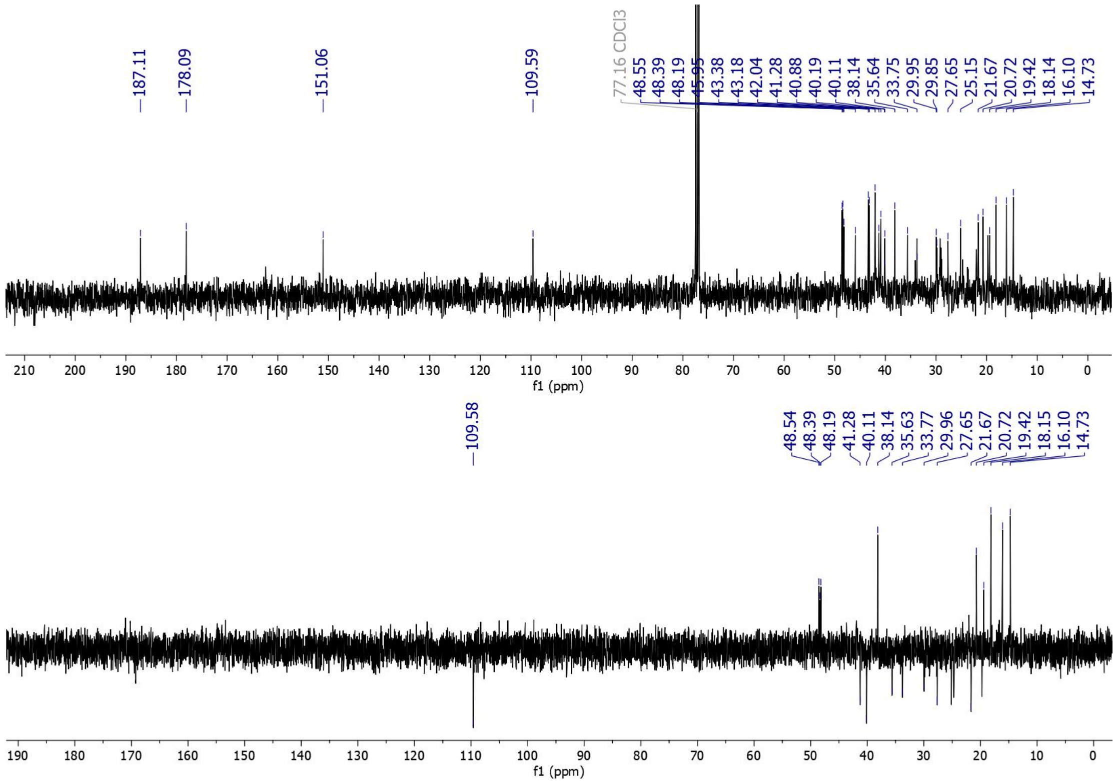

Figure S30. ^{13}C NMR and DEPT-135 spectra (100 MHz, $CDCl_3$) of compound **9**.

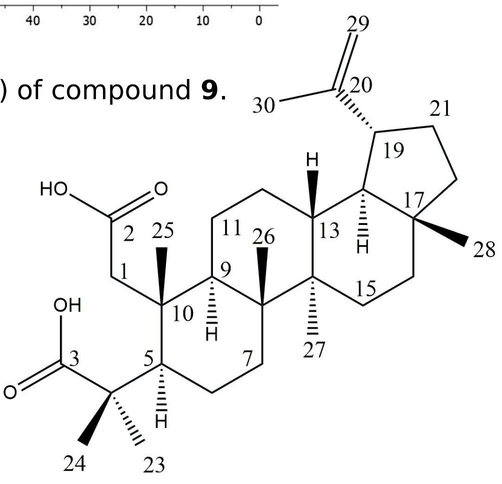

SMILES and Radar Maps.

SMILE Compound 1:

[H][C@]12[C@@H](CC[C@]1(C)CC[C@]1(C)[C@]2([H])CC[C@]2([H])[C@@](C)(CC(O)=O)[C@@]([H])(CC[C@@]12C)C(C)(C)C(O)=O)C(=C)C(C)=O

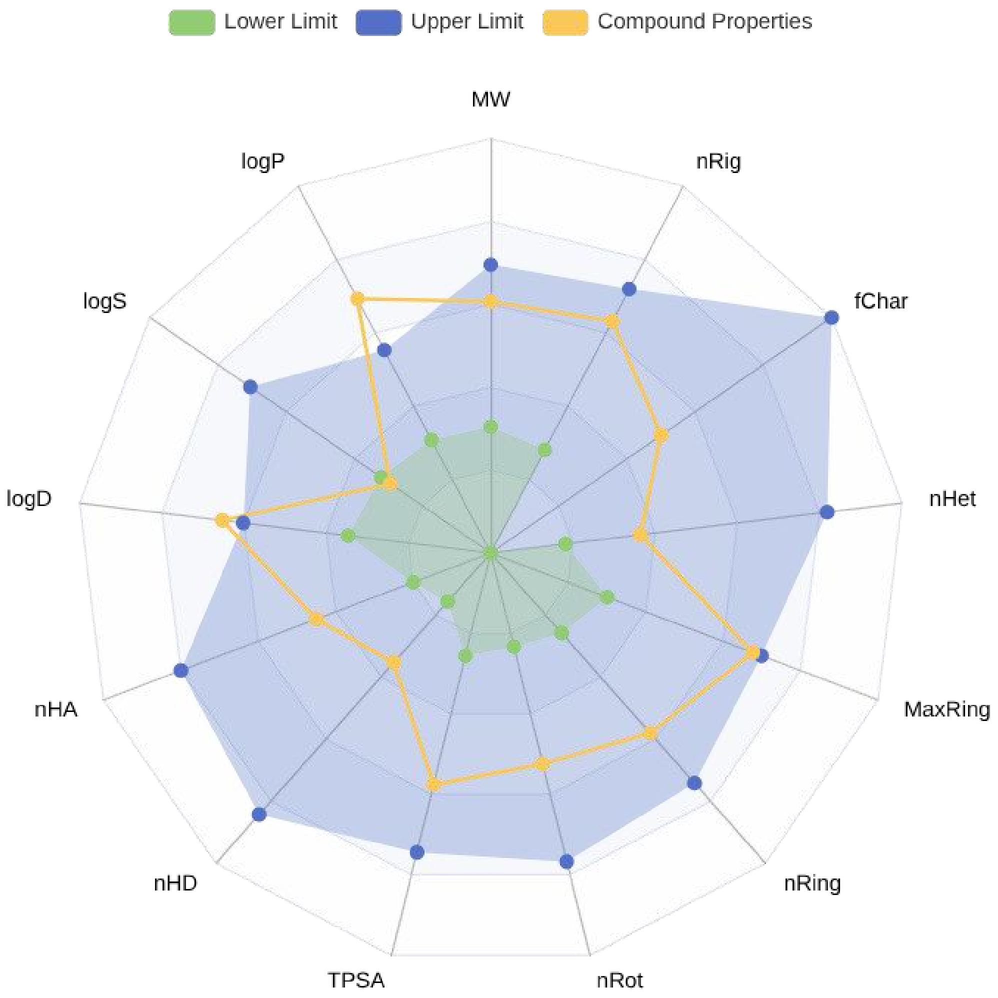

Figure S31. Radar Map obtained from ADMETlab 3.0 of compound **1**.

SMILE Compound 9:

[H][C@]12[C@@H](CC[C@]1(C)CC[C@]1(C)[C@]2([H])CC[C@]2([H])[C@@](C)(CC(O)=O)[C@@]([H])(CC[C@@]12C)C(C)(C)C(O)=O)C(C)=C

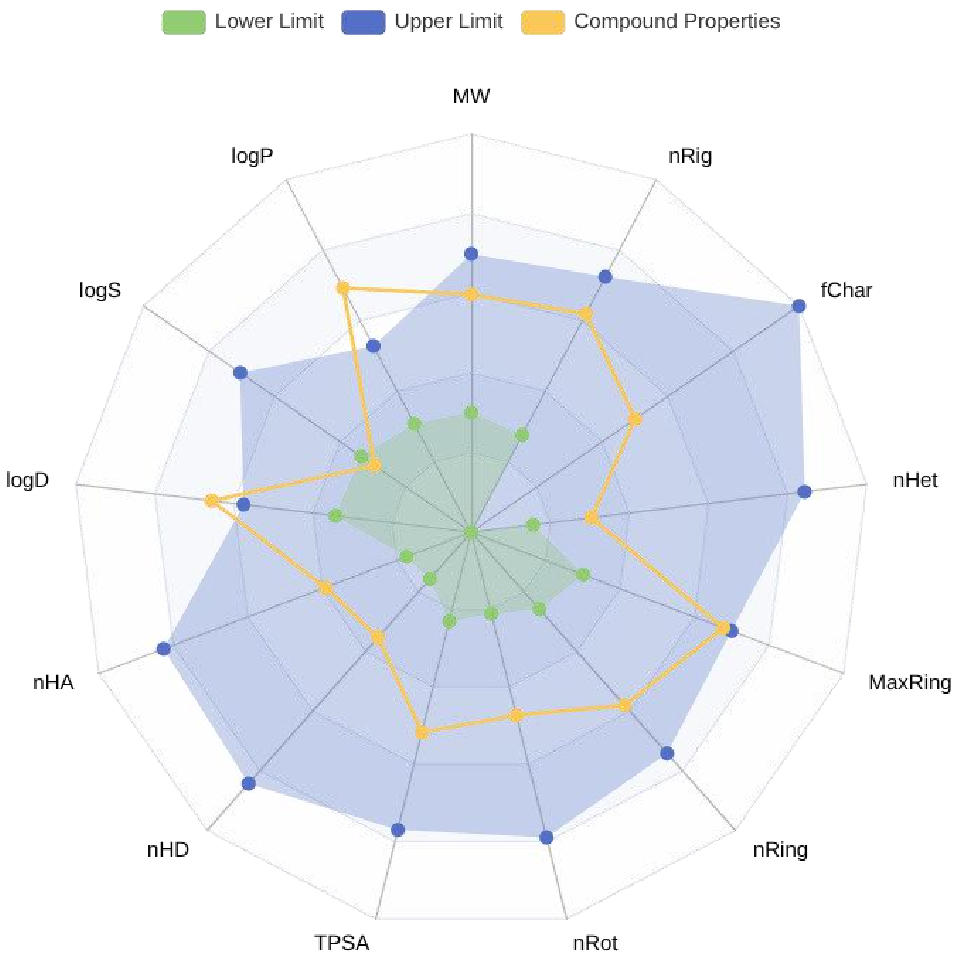

Figure S322. Radar Map obtained from ADMETlab 3.0 of compound **9**.

SMILE Lupeol

[H][C@]12[C@@H](CC[C@]1(C)CC[C@]1(C)[C@]2([H])CC[C@]2([H])[C@@]3(C)CC[C@H](O)C(C)(C)[C@]3([H])CC[C@@]12C)C(C)=C

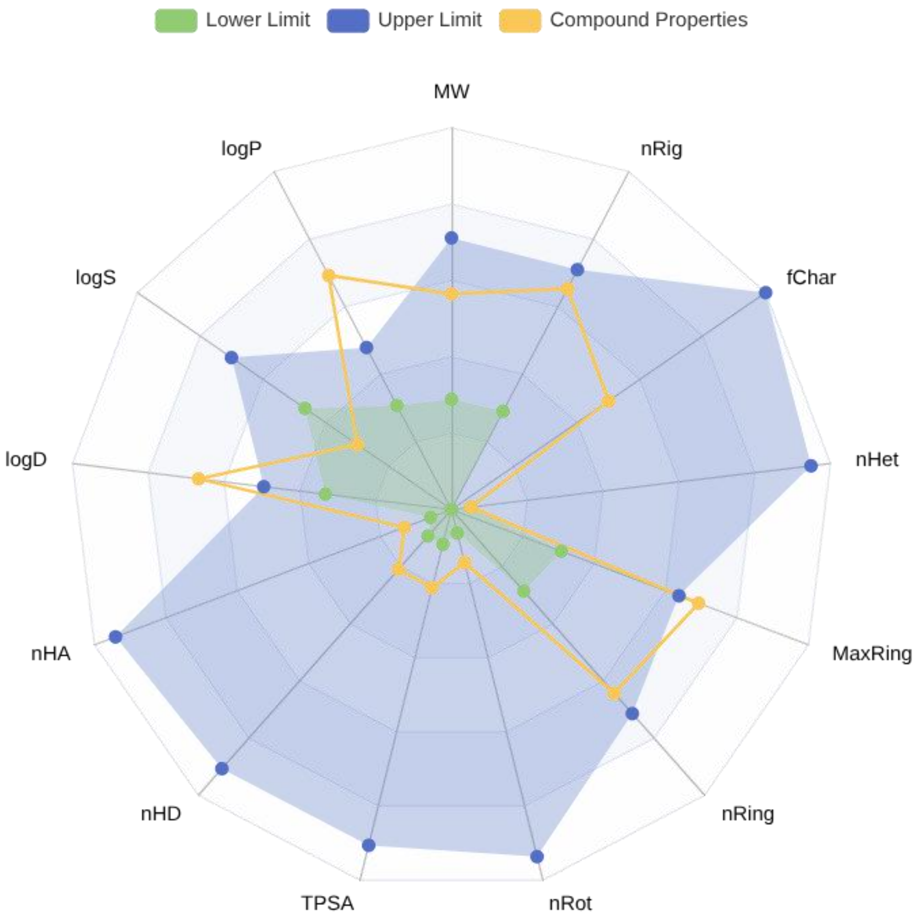

Figure S33. Radar Map obtained from ADMETlab 3.0 of **Lupeol**.

Unannotated (raw) NMR spectra of compounds **1** and **2**.

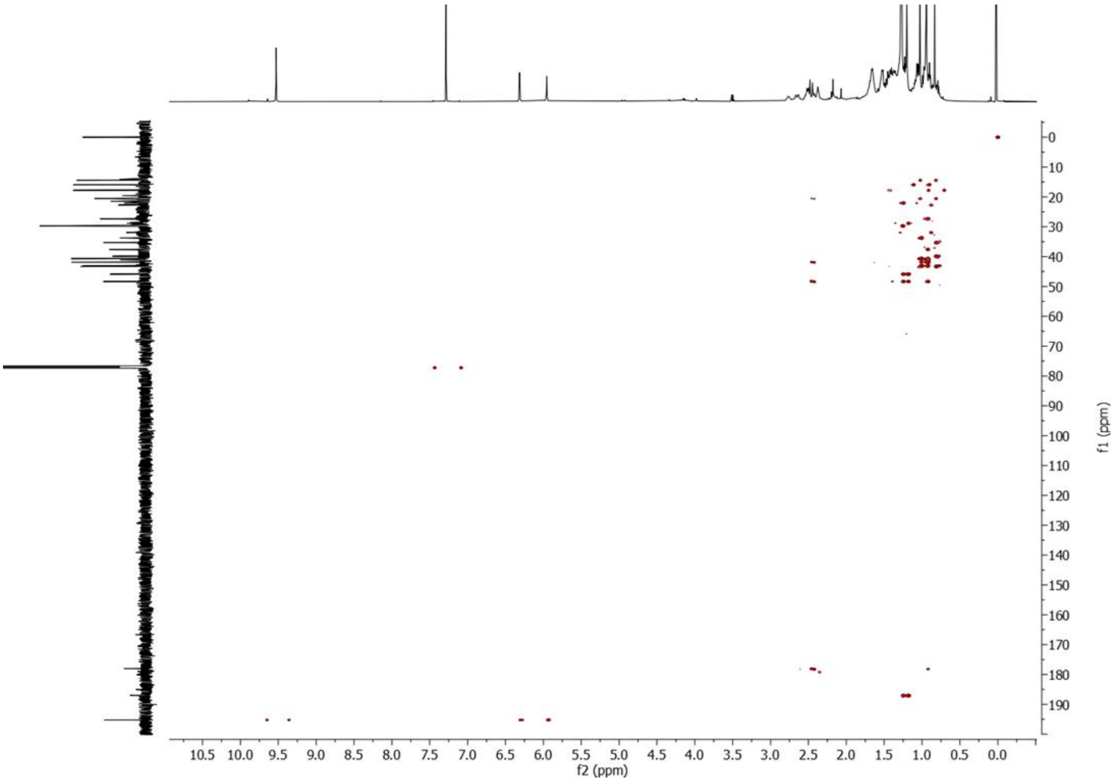

Figure S34. HMBC spectrum (600 MHz, $CDCl_3$) of compound **1.**

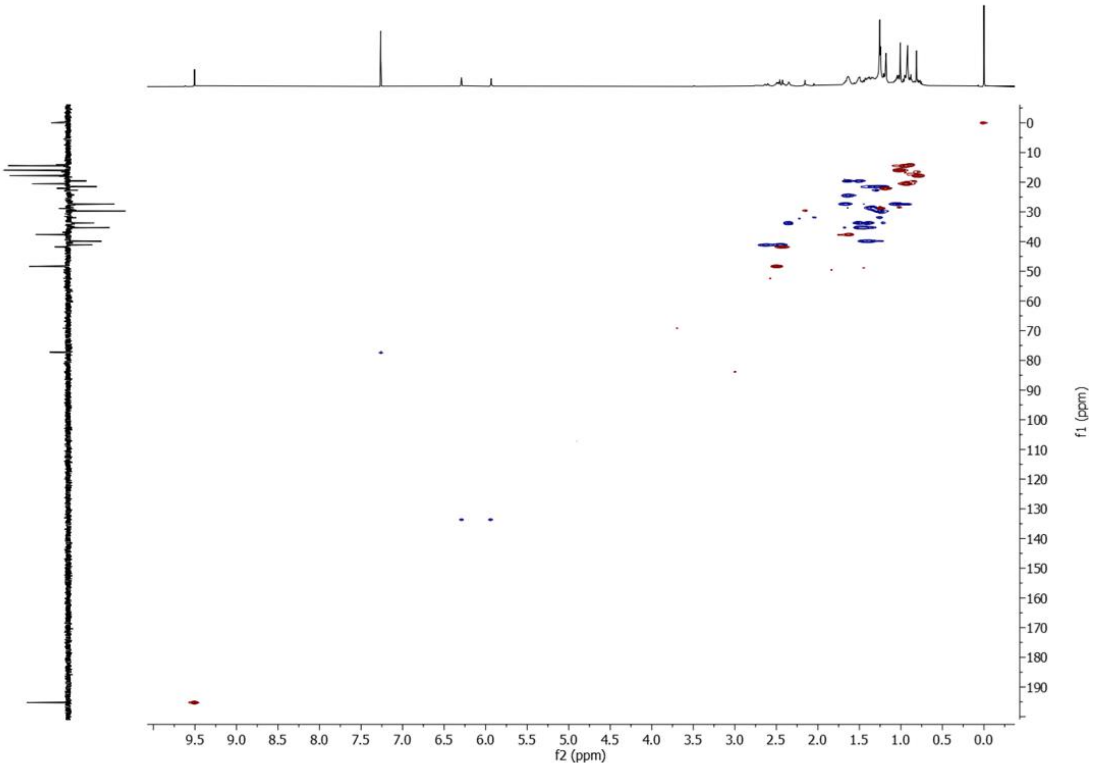

Figure S35. HSQC spectrum (600 MHz, $CDCl_3$) of compound **1.**

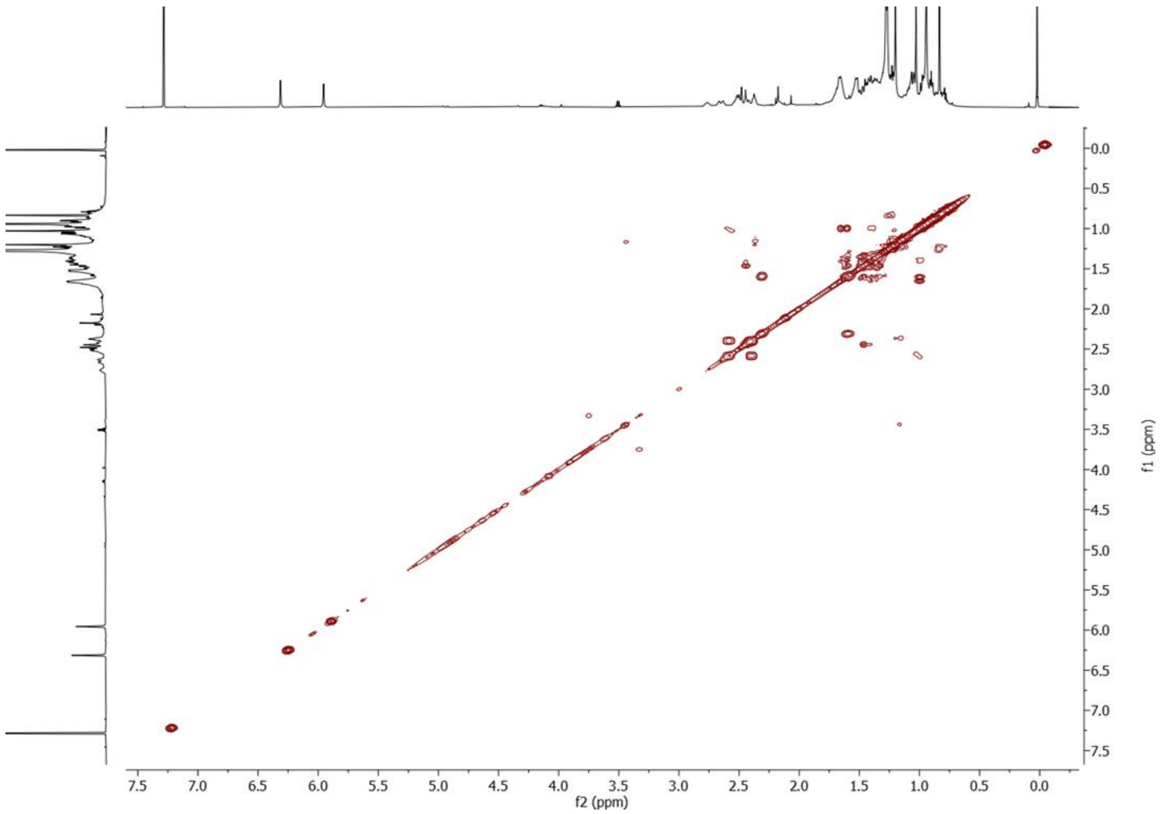

Figure S36. COSY spectrum (600 MHz, $CDCl_3$) of compound **1.**

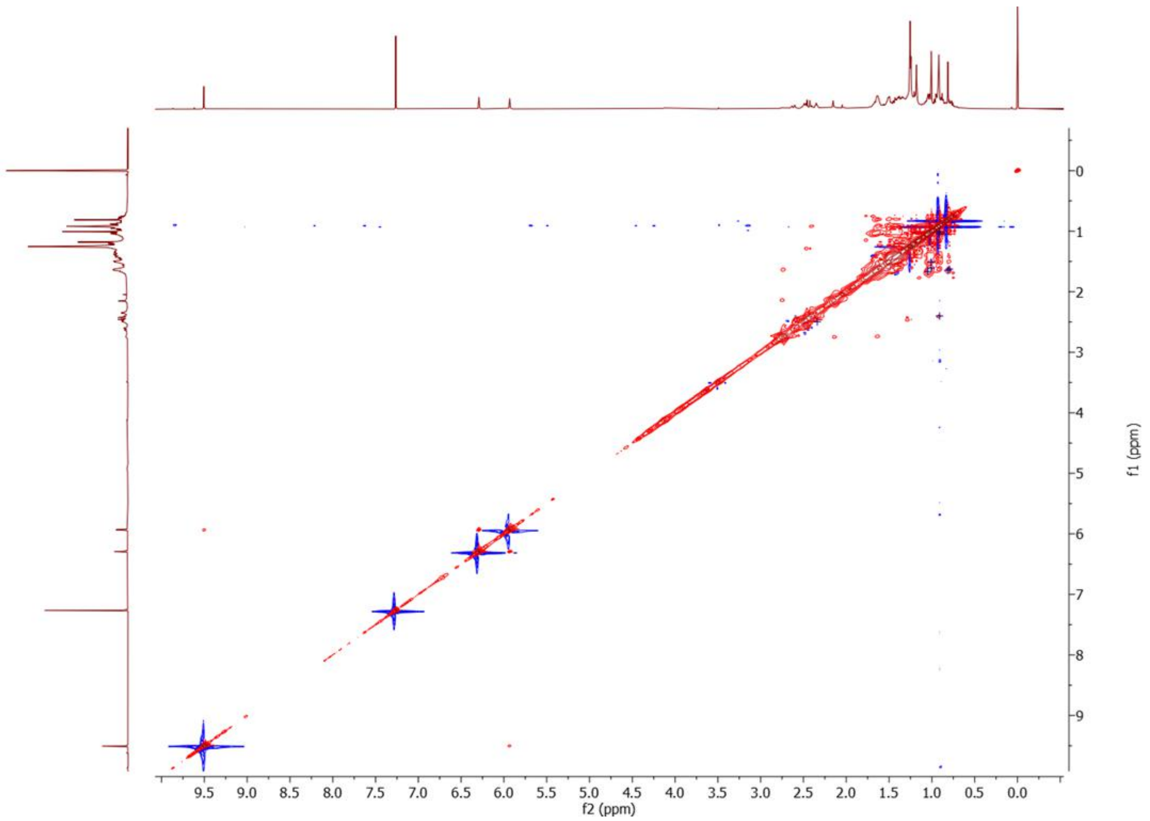

Figure S37. NOESY spectrum (600 MHz, $CDCl_3$) of compound **1.**

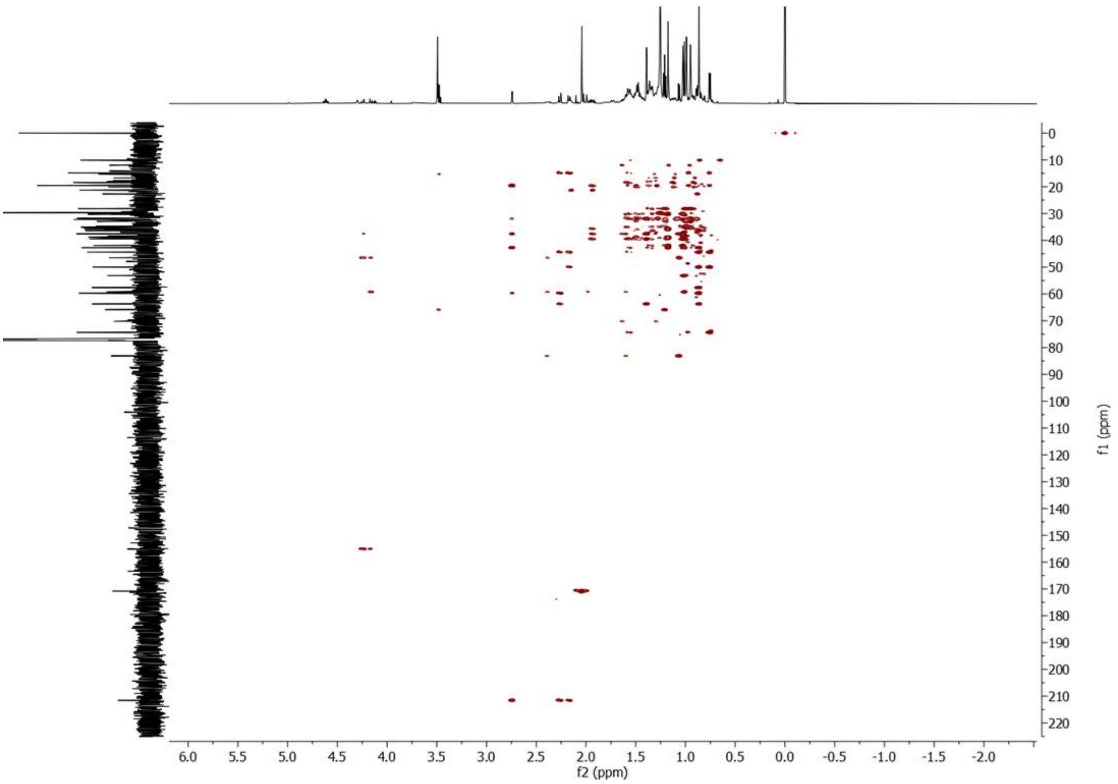

Figure S38. HMBC spectrum (600 MHz, $CDCl_3$) of compound **2.**

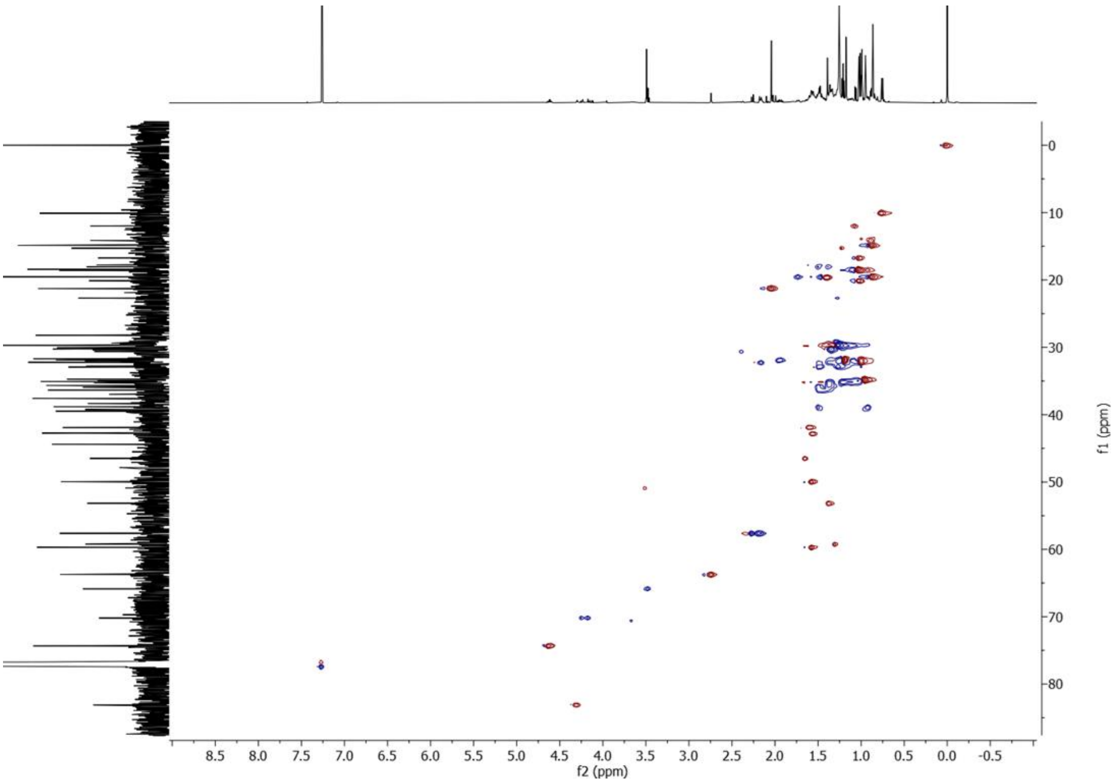

Figure S39. HSQC spectrum (600 MHz, $CDCl_3$) of compound **2.**

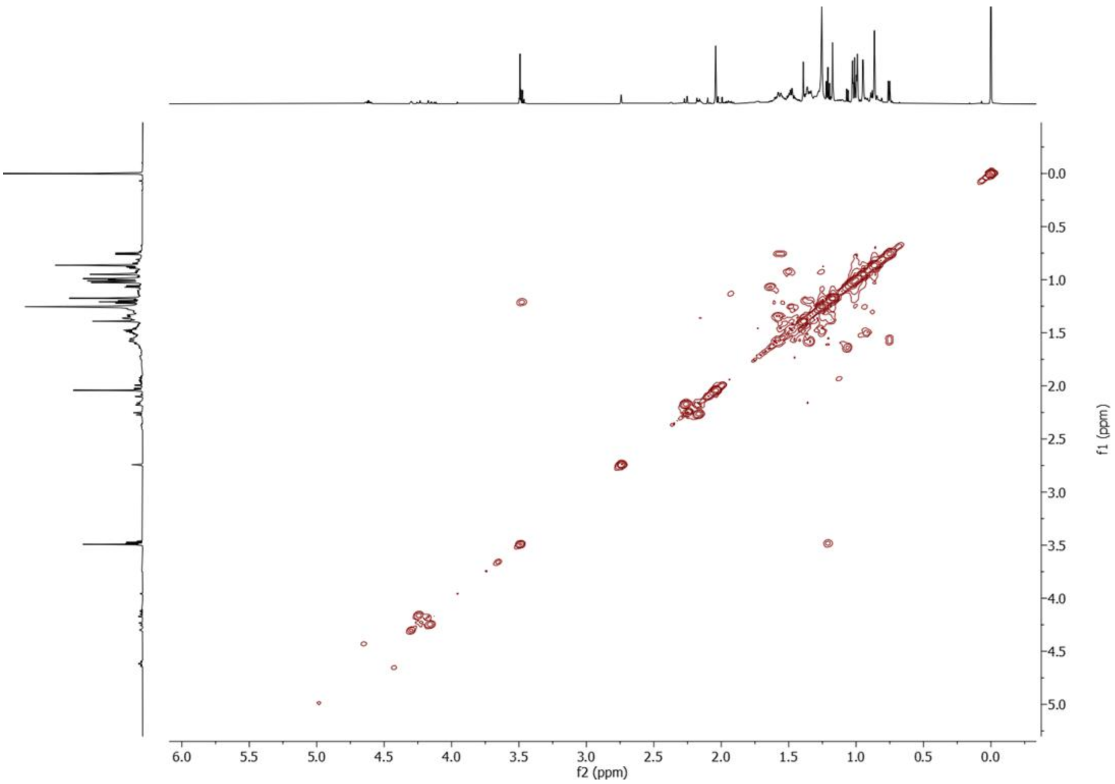

Figure S40. COSY spectrum (600 MHz, $CDCl_3$) of compound **2.**

References

1. Mahato, S. B.; Kundu. A. P. *Science,* **1994,** 37, 1517.

2. Salazar, G. C. M. *et al*. *Magnetic Resonance in Chemistry*, **2000**, 38, 977.

3. Parmar, V. S. *et al*. *Phytochemistry*, **1998**, 49, 1069.

4. Raga, D. D. *et al*. *Verlag der Zeitschrift für Naturforschung*, **2011**, 235.

5. Patra, A.; Chaudhuri. S. K. *Magnetic Resonance in Chemistry*,

1987, 25, 95.

6. Camargo, K. C.; *et al*. *Molecules,* **2022**, *27*, 959.
